# Comparative evaluation of methods to determine intra‐individual reference ranges in nutrition support team (NST)‐related tests

**DOI:** 10.1002/jcla.23639

**Published:** 2020-10-27

**Authors:** Yoji Hirabayashi, Yutaka Tsukada, Takuya Sakurai, Hideki Ohno, Takako Kizaki

**Affiliations:** ^1^ Clinical Laboratory Testing Division SRL Hachiohji Laboratories SRL, Inc Tokyo Japan; ^2^ Department of Molecular Predictive Medicine and Sport Science School of Medicine Kyorin University Tokyo Japan; ^3^ Social Medical Corporation The Yamatokai Foundation Tokyo Japan

**Keywords:** Bayesian inference, individual reference range, nutrition indicator marker, reference change value

## Abstract

**Background:**

The intra‐individual reference range is generally narrower than the commonly used reference range. Consequently, close monitoring of changes in the laboratory test results of individuals based on the inter‐individual reference range remains challenging.

**Methods:**

We examined the determination of individual reference ranges using four indicators of nutritional conditions: transferrin (TRF), albumin (ALB), retinol‐binding protein (RBP), and transthyretin (TTR). The subjects comprised 20 healthy individuals and blood samples were collected and tested five times at 2‐week intervals. We used the measurement results for the four indicators and examined individual reference ranges using four methods, including calculation methods based on the reference change value and Bayesian inference.

**Results:**

The resulting intra‐individual reference ranges were narrower than the currently used inter‐individual reference range for all measurements using four methods. Furthermore, the intra‐individual coefficient of variation [CV (intra)] was smaller than the inter‐individual coefficient of variation [CV (inter)] for TRF, RBP, and TTR for all 20 subjects. The means CV (intra) for the four indicators were also lower than the corresponding CV (inter).

**Conclusions:**

The intra‐individual reference range can be used to validate the standard deviation and coefficient of variation for currently used indicators. Moreover, Bayesian methods are speculated to be the most versatile.

## Graphical abstract

In our paper, we demonstrate a novel method of diagnostic threshold estimation using the test results of the subjects from this study. In current clinical practice, the inter‐individual reference range of analytes, such as transferrin, is used for patient diagnosis in routine clinical settings. However, in some subjects, even if the variation of the measured analyte is small, one or more of its values may surpass the normal range and be sent for reanalysis (upper diagram). In addition to two previously studied methods for constructing intra‐individual reference ranges, our current study reports on intra‐individual reference ranges that we constructed using the Bayesian estimation method (lower diagram). This new method uses the standard deviation of an individual’s daily test results taken prior to the initial diagnostic assessment to calculate the reference standard range to be used when the subject takes the diagnostic test for the first time; the reference standard range is recalculated using Bayesian estimation after the second test. It is a method to refer to the reference range obtained by the method. Clinicians will be able to diagnose patients based on the currently used inter‐individual reference range, as well as the reference range obtained by the Bayesian estimation method

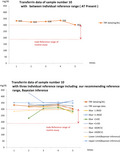

## 1 INTRODUCTION

The interpretation of laboratory results generally uses the 95% confidence interval of the distribution of test results obtained from reference individuals selected under certain conditions. This reference range is interpreted as a combination of inter‐individual and intra‐individual variations. However, there is often a gap in the mean values between the intra‐individual and the inter‐individual reference ranges. In many cases, the standard deviation of the intra‐individual reference range is smaller than the inter‐individual. Therefore, clinically significant variations may be overlooked if using the inter‐individual reference range alone. Also, observed variation, which may not be clinically significant, in the intra‐individual reference range, could be outside the inter‐individual reference range.^1^, ^2^, ^3^, ^4^, ^5^, ^6^, ^7^, ^8^ Consequently, close monitoring of variations in the clinical laboratory results of an individual is difficult and can lead to unnecessary secondary examinations if the values obtained exceed the general reference range.^1^, ^2^, ^3^, ^4^, ^5^, ^6^, ^7^, ^8^ For example, serum enzymes, such as γ‐glutamyl transpeptidase and alkaline phosphatase, in addition to uric acid, total cholesterol, and albumin (ALB), have narrower intra‐individual variations than inter‐individual variations; thus, the reference range is consistent with inter‐individual variations. Therefore, although there are significant changes in the values for an individual, such changes will not be detected as long as each is within the reference range, which is the reason for the low sensitivity of these reference ranges.^4^, ^9^

Both the intra‐individual and inter‐individual reference ranges can be used for evaluation, although there are differences in the mean value and standard deviation. If each individual subject is healthy, the measured values fall within the statistically established intra‐individual reference range.^1^, ^2^, ^3^, ^4^, ^5^, ^6^, ^7^, ^8^ However, if the measured value is statistically abnormal, an alarm can be triggered sooner than determining it with the intra‐individual reference range. This is because the intra‐individual reference range is narrower than the inter‐individual reference range.^1^, ^2^, ^3^, ^4^, ^5^, ^6^, ^7^, ^8^ Clinical physicians can use both the inter‐individual and the intra‐individual reference range to classify subjects who are undergoing a health examination/ patients who are visiting clinic as “healthy”, “observation is required” or “close examination is required” and to make a comprehensive decision, in combination with their medical knowledge and experience, if active treatment is necessary for them by using the test results as important information.

Patients suffering from malnutrition tend to suffer for an extended period of time during which their condition typically worsens.^10^, ^11^ Nutritional state management is a factor associated with metabolic disorders and slow healing of wounds, resulting in prolonged hospital stays.^12^ Therefore, objective nutritional evaluation (objective data assessment: ODA) is essential for patients requiring close monitoring of their nutritional state. This study examined the determination of individual reference ranges for four nutritional indicators: transferrin (TRF), ALB, retinol‐binding protein (RBP), and transthyretin (TTR).^13^, ^14^ These indicators are also biomarkers with different half‐lives. Each indicator can be determined using four methods. In Method (I), the standard deviations obtained from multiple measurements are considered as the standard deviation of the indicator, and the reference range is calculated as the mean ± 1.96 standard deviation measured for an individual.^1^, ^2^, ^3^, ^4^, ^5^, ^6^, ^7^, ^8^ Method (II) uses the reference change value (RCV), which is calculated from the mean and the coefficient of variation (CV) of measurements obtained from an individual over time, ie, the standard deviation (RCV) = RCV × mean ×1/100 is calculated and then used in the reference range = mean ±standard deviation (RCV).^15^, ^16^, ^17^ In Method (III), assuming that the individual reference range has a normal distribution, we define the range that includes 95% of the healthy measurement results as μ ± 2σ, estimated as mean ± 2S, and consider mean X − Cn (Cn = t_n−1_(0.025)√(n + 1)/n, t_n−1_(0.025): the top 2.5% of 5 distributions for freedom n − 1) < mean X < mean X + Cn as the reference range.^18^ Method (IV) is a reference range model in which variables are converted to present the measured values in a normal distribution. We first estimate inter‐individual variations, intra‐individual variations, and time effects in a mixed‐effect model that uses measured values as the response variables, the individual as the random effects, and the point‐in‐time as fixed effects (or random effects). Next, the distributions of the measured values for an individual observed during medical examinations are estimated on the basis of Bayesian inference posterior distribution with inter‐individual variations, intra‐individual variations, and overall mean as prior distributions.^19^, ^20^, ^21^, ^22^, ^23^

## 2 MATERIALS AND METHODS

### 2.1 Devices and reagents

ALB was measured with a BM8060 automated biochemical analytical device (JEOL Ltd., Tokyo, Japan) using pure‐auto S ALB reagent (KAINOS Laboratories, Inc, Tokyo Japan). TRF, RBP, and TTR were also measured with the same device using N‐assay TIA Tf‐H NITTOBO, N‐assay LA RBP NITTOBO, and N‐assay TIA prealbumin NITTOBO B‐type R‐1/R‐2 (NITTOBO MEDICAL Co., Ltd., Tokyo, Japan) as reagents.

### 2.2 Subjects

Analysis of the individual reference ranges was conducted using data measured from 20 staff members of the SRL Diagnostics Pathology Laboratory (Tokyo, Japan). These volunteers [age: 45.2 ± 8.0 years (mean ± standard deviation)] presented no abnormal findings in in‐house examinations, during interviews with an industrial physician, and had normal chest X‐rays. The volunteers comprised 11 men (age: 46.4 ± 8.1 years), of which two were in their 30s, five in their 40s, and four in their 50s, and nine women (age: 43.7 ± 8.0 years), of which three were in their 30s, three in their 40s, and three in their 50s. These subjects were healthy adults and their dietary and exercise habits were not regulated.^2^ All laboratory results were anonymous but linkable.

This study was conducted with strict adherence to the ethical policy on medical research involving humans, with approval from the SRL ethics review committee (approval no. 12‐06). All participants provided written informed consent prior to participating in the study.

### 2.3 Measurement period

We followed instructions from each diagnostic kit company when measuring the corresponding test indicator. Fasting blood samples were obtained at 8:30‐9:30 AM on five separate days at 2‐week intervals (December 13 and 27, 2012; January 10 and 24, 2013; and February 4, 2013), and tests were performed on the centrifuged serum samples on the sampling day.

### 2.4 Statistical analysis

The four test indicators were measured five times for 20 subjects. We obtained the mean, standard deviation, inter‐individual coefficient of variation [CV (inter)], and intra‐individual coefficient of variation [CV (intra)] of the measurements from each individual over time. Using the mean of five measurements obtained for each subject, we calculated the mean and standard deviation for all 20 subjects and then examined the difference among the means of the subjects with the overall mean using the F‐test and *t* test.

### 2.5 Determining the individual reference range

#### 2.5.1 Method (I): This calculation method uses the mean ± 1.96 standard deviation based on multiple measurements and the standard deviation from each individual

We obtained the mean, standard deviation, and CV for each set of measurement four results as follows, 1st and 2nd data, from 1st to 3rd data, from 1st to 4th data, and from 1st to 5th data. We considered the standard deviation obtained for measurements from 1st to 5th as the standard deviation of the test indicators, and calculated the reference range for each individual as the mean ± 1.96 standard deviation.^1^, ^2^, ^3^, ^4^, ^5^, ^6^, ^7^, ^8^

We calculated the skewness and kurtosis obtained from each item and each measured value in advance, and confirmed the normal distribution.

#### 2.5.2 Method (II): This calculation method uses CV and RCV

We calculated the mean and CV of measurements from each individual obtained over time. RCV was calculated as follows: RCV ＝ 2^(1/2)*Z*((CVa^2)+(CVi^2))^(1/2)), where Z = 1.96 (95%) or 2.58 (99%), Cva = measurement error, and Cvi = intra‐individual variations.^15^, ^16^, ^17^ In this manner, we obtained the mean after the second measurement. We calculated the standard deviation (RCV) = RCV × mean ×1/100 and used the reference range = mean ±standard deviation (RCV).

#### 2.5.3 Method (III): This calculation method uses measurement results of past normal time under the normal distributional assumption

Assuming that the individual reference range has a normal distribution, we defined the range in which 95% of the measurement results from healthy subjects fall within μ ± 2σ and estimate the error as the mean ± 2S. The smaller the number of measurements, the larger the estimation error. We determined the range in which the present measurement result X can be determined to be within or beyond the reference range based on previous measurements from healthy individuals (X1, X2, …, Xn). The present measurement results X are samples from the normal distribution population N (μ, σ^2) (μ and σ are unknown), which is equivalent to previous measurement results from healthy subjects (X1, X2, …, Xn). This is a test of the null hypothesis, and the same concept as the t test for the difference between two groups can be applied.

The T‐distribution becomes T = X‐mean (n)/S^((1＋(1^n)). If the top 2.5% of the t‐distribution with freedom n − 1 t(n − 1)(0.0025) is used, when |T|>t(n－I) (0.025), the null hypothesis is rejected with a significance level of 5%, and it can be assumed that the physiological state changed due to a certain factor. In other words, we can consider the range in which the present data have a 5% false‐positive rate, and the null hypothesis cannot be rejected (mean X–Cn < X<X mean X + Cn), as the reference range.^18^ In this study, Cn = t(n－1) (0.025) ^((n + 1)/n). Using the value of 3.041 when n = 5 as the significance level α = 0.05.^18^

#### 2.5.4 Method (IV): This calculation method uses Bayesian inference

We assumed that the individual reference ranges obtained using Methods (I)–(III) have a normal distribution of individual measurement results near the intra‐individual reference range. However, the measurement results of an individual after an infinite number of measurements will be closer to the inter‐individual mean than each individual measurement. As measurement values for an individual accumulate, the individual reference range calculated for each new measurement approaches the mean of measurement values for the individual. If we consider a value that falls outside the 95% confidence interval of the reference range obtained in this manner as abnormal, an abnormal finding for an individual can be detected earlier. A low or high baseline (the mean of measurements taken at each sampling) for an individual will not be considered abnormal, allowing false‐positives to be eliminated. The individual reference range was thus obtained with the following procedures^19^, ^20^, ^21^, ^22^, ^23^:

1. Variables were converted so that the measured values had a normal distribution.
2. A mixed effects model that used measured values as the response variables, the individual as random effects, and the point‐in‐time as fixed effects (or random effects), allowed estimation of inter‐individual variations, intra‐individual variations, and time effects.
3. Using these inter‐individual variations, intra‐individual variations, and overall mean as prior distributions, we examined a reference range model that estimates the distribution of measured values for an individual by observation based on the posterior distribution of Bayesian inference.

We assume that the mean μ0 and standard deviation σ0 of the test value X for data from a healthy subject have a normal distribution.

The test value of an individual i at time j, X_ij_, is expressed with the following equation:

(2) $$X_{\mathrm{ij}}=\mu_{i}+t_{\mathrm{ij}}+e_{\mathrm{ij}}$$

where μi is the mean of the test result X for individual i through the time j = 1, n, where t_ij_ is the temporal variation of intra‐individual test values and e_ij_ are measurement errors.

Let us assume that t_ij_ and e_ij_ have the same dispersions τ^2^ and σε^2^ regardless of the time and subject, and τ and ε are independent.

Then, the test value X_ij_ follows a normal distribution N (μi, τ^2^ + ε^2^).

Both τ^2^ and σε^2^ are known and assumed to be σ^2^ = τ^2^ + ε^2^.

The population mean μ0 and standard deviation σ0 are predicted ahead of time, and with this prior distribution, the mean for N observations, and assuming a dispersion of σ2, follows a normal distribution of N (μn, σn) using Bayesian interference.

If.

(3) $$\mu_{n}=\frac{\sigma^{2}}{n\sigma_{0}^{2}+\sigma^{2}}\mu_{0}+\frac{\sigma_{0}^{2}}{n\sigma_{0}^{2}+\sigma^{2}}\sum_{j=1}^{n}X_{j}$$

(4) $$\frac{1}{\sigma_{n}^{2}}=\frac{1}{\sigma_{0}^{2}}+\frac{n}{\sigma^{2}}$$

or

$$\sigma_{n}^{2}=\frac{\sigma^{2}\sigma_{0}^{2}}{\sigma^{2}+n\sigma_{0}^{2}}$$

then individual reference values are assumed to have a distribution of μn estimate errors, temporal variations, and measurement errors of intra‐individual examinations around the individual mean μn. Assuming that these errors have a normal distribution and are independent of each other, the lower and upper limits of individual reference values with n measurements are expressed as follows:

$$\text{Lower}\;\text{limit}=\mu_{n}‐1.96\times \left(\frac{\sigma^{2}\sigma_{0}^{2}}{\sigma^{2}+n\sigma_{0}^{2}}+\sigma^{2}\right).$$

$$\text{Upper}\;\text{limit}=\mu_{n}+1.96\times \left(\frac{\sigma^{2}\sigma_{0}^{2}}{\sigma^{2}+n\sigma_{0}^{2}}+\sigma^{2}\right).$$

If there is no observation, the reference value for the test is the individual reference value.

### 2.6 Analytical accuracy

We obtained acceptable accuracy for the four target indicators with the mean X‐Rs‐R method with two kinds of reference sera during the measurement period (December 2012, January 2013, and February 2013). The reference sera were L‐Consela 1EX (Lot No. 079 207) and L‐Consela 2EX (Lot. No. 142 207) for ALB and TRF, and Immuno‐quest L‐1 (Lot No. A243A) and Immuno‐quest L‐II (Lot No. K251A) for RBP and TTR. We obtained the mean total variations for the total variations in each reference serum in monthly sets collected over a 2 month period.

## 3 RESULTS

### 3.1 Analytical accuracy

The mean total variations during the measurement period were TRF = 2.87, ALB = 0.12, RBP = 0.05, and TTR = 0.54, whereas the CVs were TRF = 1.07%, ALB = 2.88%, RBP = 1.38%, and TTR = 1.83% (Table 1).

**Table 1:** Total variation of 4 items during measurement period

Item abbreviation (Unit)	Control Sample	Total Variation	
TRF (mg/dL)	L‐Consera1EX/2EX	SD	2.87
		CV(%)	1.07
ALB (g/dL)	L‐Consera1EX/2EX	SD	0.12
		CV(%)	2.88
RBP (mg/dL)	Immuno‐Quest L‐Ⅰ/Ⅱ	SD	0.05
		CV(%)	1.38
TTR (mg/dL)	Immuno‐Quest L‐Ⅰ/Ⅱ	SD	0.54
		CV(%)	1.83

### 3.2 Determining the reference range by each statistical analysis method

**Figure 1:**
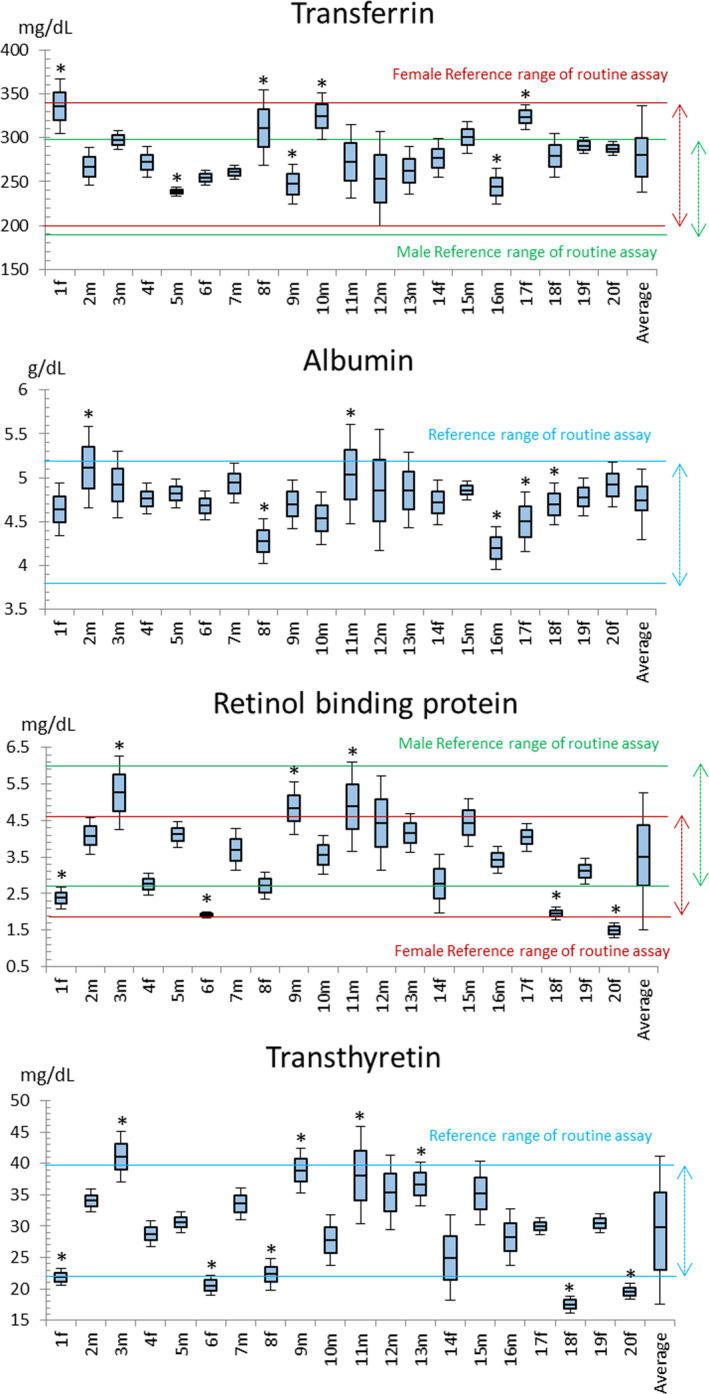
Mean and standard deviation and etc for measurements of four test indicators for each subject. The mean and standard deviation (SD) were calculated for five measurements of transferrin, albumin, retinol‐binding protein, and transthyretin for each of the 20 subjects, along with the mean and standard deviation for all 20 cases. The blue box with black lines shows the box‐plot figure consisted of the mean value, the mean + 2SD value, the mean −2SD value, the minimum value, and the maximum value for each subject. *P < .05 vs. average. On x‐axis, 1f, 4f, 6f, 8f, 14f, 17f, 18f, 19f, and 20f mean the female subjects. On the other hand, 2m, 3m, 5m, 7m, 9m, 10m, 11m, 12m, 13m, 15m, and 16m mean the male subjects. Furthermore, “Average” on x‐axis is calculated by each test indicator for the 20 subjects. On y‐axis, the values indicate the concentrations of each test indicator. The range among two green lines mean the male reference range of routine assay (see Result 3.2), the range among two red lines mean the female reference range of routine assay (see RESULTS 3.2), and the range among two light blue lines show the total (no gender differences) reference range of routine assay (see RESULTS 3.2)

We obtained the maximum, minimum, mean, and standard deviation for the four indicators measured in 20 subjects based on five measurements per subject. Figure 1 compares the results for each test indicator for the 20 subjects and the results of the five measurements for each subject. The data were statistically analyzed using the four methods described above and the following results were obtained: Method (I), mean ± 1.96 standard deviation; Method (II), mean ± standard deviation (RCV); and Method (III), mean ± 3.041 standard deviation (mean ± 2S) (Table 2). The RCV obtained from the results of five measurements for each test indicator were as follows: TRF = 11.64%, ALB = 12.68%, RBP = 20.54%, and TTR = 15.89% (Table 2). We also performed a statistical analysis with Bayesian inference^14^, ^19^, ^20^, ^21^ [Method (IV)] (Table 3). Reference ranges for each test indicator shown in Table 3 and Figure 1 were as follows: TRF = 190‐340 mg/dL (men: 190‐300 mg/dL, women: 200‐340 mg/dL),^24^ ALB = 3.8‐5.2 g/dL,^25^ RBP = 2.7‐6.0 mg/dL (men: 2.7‐6.0 mg/dL, women 1.9‐4.6 mg/dL),^26^ and TTR = 22.0‐44.0 mg/dL.^18^

**Table 2:** Comparison of current reference range and reference range calculated by three methods

Item abbreviation (Unit)	Results of statistical data analysis of five measurements of 20 subjects to be verified within this time range						RCV(%)	Xbar ± 1.96SD (Results of this study)		The difference between the upper limit and the lower limit	Xbar ± SD(RCV) (Results of this study)		The difference between the upper limit and the lower limit	Xbar ± 3.041SD (Results of this study)		The difference between the upper limit and the lower limit
	Max.	Min.	Between the maximum value and the minimum value difference	Average	standard deviation	CV (%)										
TRF (mg/dL)	336	239	97	280	28.3	10.1	11.64	225	336	111	248	312	64	231	329	98
ALB (g/dL)	5.1	4.2	0.9	4.7	0.23	4.9	12.68	4.3	5.2	0.9	4.2	5.3	1.1	4.3	5.2	0.9
RBP (mg/dL)	5.3	1.5	3.8	3.5	1.08	30.9	20.54	1.4	5.6	4.2	2.7	4.3	1.6	3.1	4.0	0.9
TTR (mg/dL)	41.1	17.5	23.6	29.8	6.93	23.2	15.89	16.2	43.4	27.2	25.0	34.7	9.7	27.2	32.0	4.8

**Table 3:** Estimation of inter‐individual variability and intra‐individual variation using mixed effects model

Item abbreviation (Unit)		Dispersion	Standard Deviation	Total	Reference Range used by current routine assay		
				standard deviation	SEX	lower limit	Upper limit
TRF( mg/dL)	Inter‐individual variation	770.96	27.77	30.61	Male	190	300
	Individual internal transition and measurement error variation	165.95	12.88		Female	200	340
ALB (g/dL)	Inter‐individual variation	0.05	0.22	0.28	Total	3.8	5.2
RBP (mg/dL)	Inter‐individual variation	1.13	1.06	1.11	Male	2.7	6.0
	Individual internal transition and measurement error variation	0.10	0.32		Female	1.9	4.6
TTR (mg/dL)	Inter‐individual variation	47.29	6.88	7.14	Total	22.0	40.0

### 3.3 Comparison of inter‐individual CV and intra‐individual CV

**Figure 2:**
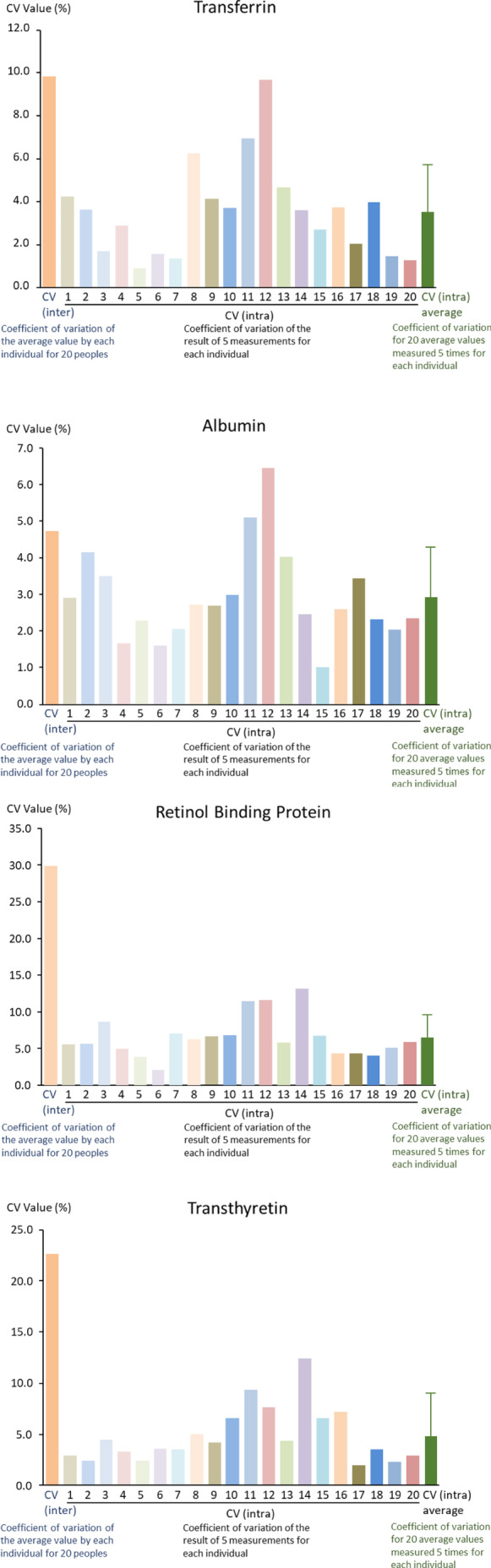
Comparison of inter‐individual and intra‐individual coefficient of variations. Coefficient of variation (CV) (inter) and CV (intra) were calculated for each measured indicator using five measurements from the 20 cases, along with the mean and standard deviation of CV (intra) for the 20 cases. On x‐axis, CV (inter) means the coefficient variation of the average value by 20 subjects. The number 1 to 20 shows the coefficient variation of the measurement data of five measurements for each subject, and CV (intra) means the average data of the CV for each subject. On y‐axis, the value shows the coefficient variation data

Next, we compared CV (inter) and CV (intra) calculated using the five measurement results for the 20 subjects (Figure 2). For TTF, RBP, and TTR, the CV (intra) was smaller than the CV (inter) for all 20 cases. For ALB, the CV (intra) was smaller than the CV (inter) in 18 of the 20 cases (Figure 2). The mean CV (intra) of the 20 subjects was lower than CV (inter) for TRF, RBP, ALB, and TTR (Figure 2).

### 3.4 Temporal variations in reference range estimates

**Figure 3:**
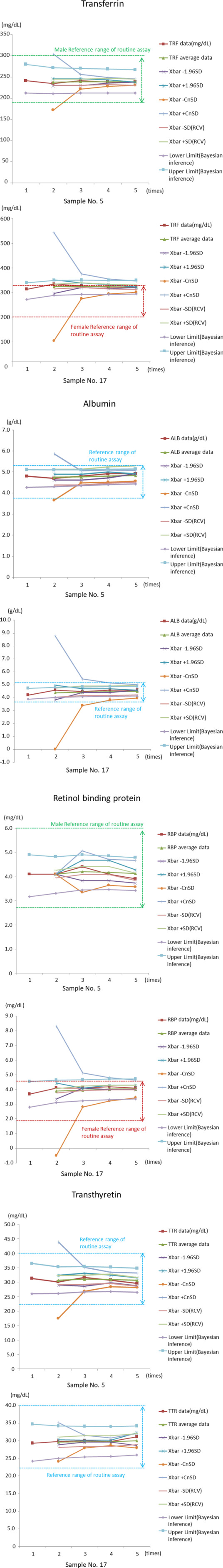
Temporal changes in measured values for the four test indicators. Temporal changes in five measurements of transferrin, albumin, retinol‐binding protein, and transthyretin for subjects No. 5 (man) and No. 17 (woman) were shown. The x‐axis shows the number of measurements. The y‐axis shows the measured concentration. The range of the red broken line shows the standard range between individuals used as the routinely analysis. The red square line shows the measured value of each time. The green ▲ line shows the cumulative average of the values measured each time. The purple x line and thin blue ⁎ line show the average value of −1.96 standard deviation (SD) and + 1.96SD, respectively. The orange line and the thin purple | line indicate the average value of ‐Cn x SD and + Cn x SD. The light red line and the light green line indicate the average value‐SD (RCV) value and average value + SD (RCV) value, respectively. Thin purple ◆ line and thin blue ■ line show the lower and upper limits of Bayesian inference, respectively. The range among two green lines mean the male reference range of routine assay (see RESULTS 3.2), the range among two red lines mean the female reference range of routine assay (see RESULTS 3.2), and the range among two light blue lines show the total (no gender differences) reference range of routine assay (see RESULTS 3.2)

Using the five measurements from the 20 subjects for the four test indicators (Supplemental Figure S1), we examined variations in the four types of reference range estimates. The results for two of the 20 subjects (Sample No. 5 and Sample No. 17) are shown in Figure 3. The first measurement was determined by comparing mean X–Cn < mean X < mean X + Cn and the reference ranges reported in previous studies.^18^, ^24^, ^27^ For reference ranges estimated using Methods [(I)–(III)], the approximation tendency could be confirmed from the third measurement. In contrast, the reference range obtained with Method (IV) was much wider than those obtained with Methods (I) and (II), whereas it was narrower than that obtained with Method (III) (Figure 3). Intra‐individual reference ranges examined with the four methods in the present study were also narrower than inter‐individual reference ranges currently being used after five measurements (Figure 3).

## 4 DISCUSSION

The typical procedure to determine a reference range is as follows^25^, ^28^, ^29^, ^30^, ^31^:

1. Reference individuals are selected from healthy individuals. A population of reference individuals selected for each sex and age group comprises at least 120 individuals.
2. Statistical analysis: mean ± 2 standard deviation (more accurately, 95% of the normal distribution is equivalent to mean ± 1.96 standard deviation, and mean ± 2 standard deviation is the range that includes 95.45% of the normal distribution).
3. The above selection conditions for reference individuals, measurement conditions, and statistical analysis must be clearly stated.

In other words, the reference range of test values is expressed as a 95% confidence interval of inter‐individual variations, including measurement errors. Medical examination data are repeatedly measured for each individual, and as new information is added to the data longitudinally (inter‐individually), distributions can be analytically divided into inter‐individual variations and other errors, ie, intra‐individual variations. The most natural interpretation of inter‐individual variations is a variable model in which individuals have normal distribution around the inter‐individual mean.^19^, ^20^, ^21^, ^22^, ^23^ In contrast, a previous study on triglyceride determined the reference range for an individual using between 25 to 7055 cases and found Cvi values ranging from 2.3% to 31.9% for the shortest measurement interval of several times a day to once every 2.5 months.^32^ Earlier studies on high‐density lipoprotein (HDL) cholesterol showed that a population ranging from 25 to 1,058 cases provided Cvi values of 4.8%–10.0% .^1^, ^2^, ^24^, ^25^, ^26^, ^33^, ^34^ The RCV for ALB was reported to be 14.5%,^35^ similar to the RCV value of 12.7% obtained in this study.

Three factors can cause variations in measured values: disease, physiology, and measurement technique.^1^, ^2^, ^26^, ^27^, ^36^ Physiological variations may include the age, sex (including pregnancy and menstrual period), and dietary factors (such as meals, drinking, smoking, and stress) of an individual, inter‐individual variations affected by genetic factors, and intra‐individual variations such as the condition of the individual prior to the examination (such as position, long‐ or short‐term exercise), and conditions associated with blood sampling, such as the time of day.^37^ In contrast, in terms of the limit of permissible errors for measurement methods, Tonks^38^ divided ¼ of the reference range by the median of the reference range as the reference to evaluate the performance of the control survey for serological components; as a result, the maximum was set at 10%. Kitamura^9^ and Cotlove et al^39^ focused on a component with intra‐individual variations much narrower than the range of variations for the population by studying the physiological variations in an individual. They proposed the limit of permissible error (CV%) = ½ × (standard deviation for physiological intra‐individual variations)/(mean of reference range) × 100. CV is expressed as CV% = standard deviation × 100/mean (%), which leads to total CV (CVt: total) = measured CV (CVa: analysis) + pre‐measurement CV (CVp: pre‐analysis) + inter‐individual CV (Cvi: individual), which are indicators of intra‐individual and inter‐individual variations.^40^

The concept of individual reference was proposed by Williams^41^ in 1967, and a long‐term evaluation of health conditions of individuals was considered to lead to the early discovery of chronic diseases. In many tests, variations caused by physiological factors were larger for inter‐individual than for intra‐individual assessments, which led to the acknowledgment of the importance of intra‐individual variations.^42^ In the current study, we examined individual reference ranges for Methods (I)–(III) and compared these with the commonly used reference range (inter‐individual reference range). We found that the individual reference ranges calculated using the three methods were narrower, closely capturing physiological variations in each individual. Furthermore, we examined Method (IV) as a new model to calculate reference ranges. Method (IV) is a mixed model of inter‐individual reference range and intra‐individual reference range, which allows calculation of a reference range for each individual while using the inter‐individual reference range routinely used in clinical settings. Consequently, Method (IV) would be easily accepted in routine clinical settings. Nevertheless, these evaluations have taken the evaluation of the first measurement and the total fluctuation into consideration and thus, in terms of applicability, it is difficult to apply on new patients and experiment subjects.

The present study examined 20 cases and we obtained good results in determining the individual reference range. Currently, the commonly used inter‐individual reference range is the mean ± 1.96 standard deviation of the reference individual. With Method (III), initially proposed by Tango,^18^ it was suggested that the reference range first obtained in almost 20 subjects was very widely calculated, and it was difficult to use as the individual reference range. The present study demonstrated that a small number of measurements leads to a high estimation error when setting the individual reference range, and that calculating standard deviation from the RCV using the method proposed by Fraser^43^ [Method (II)] is useful. In contrast, using Fraser's method, the RCV must be obtained for each item ahead of time. The method proposed by Tango^18^ is more versatile and the RCV converges after three measurements. Therefore, evaluating these methods with health examination and clinical data from actual subjects may provide information more useful in clinical settings. Nevertheless, these evaluations have taken the evaluation of the first measurement and the total fluctuation into consideration and thus, in terms of applicability, it is difficult to apply on new patients and experiment subjects.

On the other hand, since Bayesian inference can estimate individual referential area from the second measurement of new patients and subjects, its medical information is, all in all, more efficient and appropriate than the ones from conventional ways. In other words, clinical physicians might judge within daily criteria (among individuals) by using the initial values and install Bayesian inference in the system for the second time onward. By using the initial values then the measured ones since the second time onward, an integrated individual criteria area can be estimated without choosing smaller items. This Bayesian inference is a mixed model of the collective criteria area among individuals (in the initial check) and individualized criteria area (the second time onward). In the initial check, no subject has previous values so the normal criteria area in daily check is used. Then, a shift to Bayesian inference from the second time can increase the applicability. Hence, if we could install and utilize LIS (Laboratory information System) in the medical check systems in hospitals, useful information could be attained without placing extra burden on clinical physicians. Furthermore, when the individualized referential area is narrower than the collective one, changes and development of the diseases of the patients or subjects might be spotted earlier for appropriate treatments. On the other hand, when the individualized referential area is wider than the collective one, unnecessary treatments might be avoided.

## 5 CONCLUSION

In the present examination, TRF, RBP, and TTR had lower CV (intra) than CV (inter) in all 20 subjects, and the mean CV (intra) was lower than the mean CV (inter) for TRF, RBP, and TTR. In contrast, CV (intra) was higher than CV (inter) for ALB in two of the 20 cases although mean CV (intra) for ALB was lower than that of CV (inter), suggesting that there may be cases where the intra‐individual reference value is not appropriately understood. Nevertheless, the preferred method for determining the individual reference range should allow close observation of temporal changes in test indicators with large inter‐individual differences. Such methods will play an important role in the development of new biomarkers and in routine diagnosis. For nutrition support team (NST)‐related test indicators in particular, the results obtained using the chosen method should closely reflect, for example, the postoperative nutritional state, allowing management of central venous nutrition and the reduction of complications (infections), thereby closely capturing the nutritional state of an individual.^14^, ^19^ Furthermore, such methods could be widely applied to test indicators such as those related to pre‐ and post‐dialysis tests and glucose tolerance tests.

## CONFLICT OF INTEREST

There are no conflicts of interest to be disclosed.

## AUTHORS CONTRIBUTIONS

Each author has made an important scientific contribution to the study and the manuscript.

## Supporting information

Fig S1

Supplementary Material

## References

[1] Hosogaya S , Ozaki Y . Quality assurance of analytical methods to guarantee the reliability of medical decision levels for interpretation of clinical laboratory data. The Jpn J Clin Pathol. 2004;52:548‐551. 15283171

[2] Barnett RN . Medical significance of laboratory results. Am J Clin Pathol. 1968;50(6):671‐676. 5726125 10.1093/ajcp/50.6.671

[3] Fraser CG , Peake MJ . Problems Associated with Clinical Chemistry Quality Control Materials. CRC Crit Rev Clin Lab Sci.. 1980;12(1):59‐86. 6993101 10.3109/10408368009108726

[4] Kiyomi F , Nishikawa M , Yoshida Y , et al. Comparison of intra‐individual coefficients of variation on the paired sampling data when inter‐individual variations are different between measures. BMC Res Notes. 2016;19(9):115. 10.1186/s13104-016-1912-y PMC4760001 26896465

[5] Kawaguchi K , Ichihara K . Physiological variation of clinical examination items. Jpn J Med Tech. 2015;64:143‐154.

[6] McWhirter JP , Pennington CR . Incidence and recognition of malnutrition in Hospital. Br Med J. 1994;308(6934):945‐948. 8173401 10.1136/bmj.308.6934.945 PMC2539799

[7] Shinohara K , Hamasaki N , Takagi Y , et al. Japanese Committee for Clinical Laboratory Standards and the Japanese Association of Medical Technologists: Multianalyte Conventional Reference Material (MacRM): A Useful Tool for Nationwide Standardization of Laboratory Measurements for Medical Care‐A Model Study in Japan. Clin Chem. 2016;62(2):392‐406. 26667777 10.1373/clinchem.2015.245621

[8] Omar F . Essential laboratory knowledge for the clinician Laboratory testing forms an integral part of patient management. Continuing Medical Education. 2012;30:244‐248.

[9] Kitamura M , Historical K . aspects of cholesterol value by various analytical methods. The Jpn J Clin Pathol. 1991;39:495‐500. 2072571

[10] Blackburn GL , Bistrian BR , Maini BS , et al. Nutrition and metabolic assessment of hospital patient. JPEN J Parenter Enteral Nutr. 1977;1(1):11‐22. 98649 10.1177/014860717700100101

[11] Ritchie RF , Palomaki GE , Neveux LM , et al. Reference distributions for the negative acute phase serum proteins, albumin, transferrin and transthyretin: A practical, simple and clinically relevant approach in a large cohort. J Clin Lab Anal. 1999;13(6):273‐279. 10633294 10.1002/(SICI)1098-2825(1999)13:6<273::AID-JCLA4>3.0.CO;2-X PMC6808097

[12] Robinson G , Goldstein M , Levine GM . Impact of nutrition status on DRG length of stay. JPEN J Parenter Enter Nutr. 1987;11(1):49‐51. 10.1177/014860718701100149 3102782

[13] Kameko M , Ota H , Ishii K , et al. Distribution of retinol‐binding protein in the human digestive tract. Virchows Arch B Cell Pathol Incl Mol Pathol. 1992;61(5):315‐322. 1348893 10.1007/BF02890433

[14] Takagi Y . Nutritional assessment protein. The Jpn J Clin Pathol. 2004;52:361‐366.

[15] Erden G , Tezcan G , Soydas ÖA , et al. Biological variation and reference change value (RCV) of prostate specific antigen (PSA) levels in the serum of healthy young individuals. Gazi Med J. 2009;20:152‐156.

[16] Bugdayci G , Oguzman H , Arattan HY , et al. The use of reference change values in clinical laboratories. Clin Lab. 2015;61(3–4):251‐257. 25974990 10.7754/clin.lab.2014.140906

[17] Callum GF . Reference change values. Clin Chem Lab Med. 2011;50(5):807‐812. 21958344 10.1515/CCLM.2011.733

[18] Tango T . An interpretation of normal ranges based on individual difference quotient: extension to multivariate case. Med Informatics. 1982;7:119‐126. 10.3109/14639238209010705 7144324

[19] Spiegelhalter DJ , Myles JP , Jones DR , et al. Bayesian methods in health technology assessment: a review. Health Technol Assess. 2000;4(38):1‐130. 11134920

[20] Spiegelhalter DJ , Abrams KR , Myles JP . Bayesian Approaches to Clinical Trials and Health‐Care Evaluation. New York: John Wiley & Sons Ltd; 2004. ISBN-10 :0471499757

[21] Spiegelhalter DJ , Myles JP , Rones DR , et al. Methods in health service research. An introduction to bayesian methods in health technology assessment. BMJ. 1999;319(7208):508‐512. 10454409 10.1136/bmj.319.7208.508 PMC1116393

[22] Raiffa H , Schlaifer R . Applied Statistical Decision Theory. Boston: Clinton Press; 1961. ISBN-10: 0875840175

[23] Blaza T , David LD , Hugo GA , et al. A Bayesian approach to the evaluation of comparisons of individually value‐assigned reference materials. Anal Bioanal Chem. 2012;403(2):537‐548. 22389172 10.1007/s00216-012-5847-4

[24] Ichikawa K , Kawai T . Determination of reference intervals for 13 plasma proteins based on IFCC international reference preparation (CRM470) and NCCLS proposed guideline (C28‐P, 1992): a strategy for partitioning reference individuals with validation based on multivariate analysis. Establishment of reference intervals and physiological parameters for 13 serum proteins in healthy Japanese adults. J Clin Lab Anal. 1997;11:117‐124. 9058246 10.1002/(SICI)1098-2825(1997)11:2<117::AID-JCLA8>3.0.CO;2-8 PMC6760695

[25] Ichihara K , Yamamoto Y , Hotta T , et al. Committee on Common reference intervals, Japan Society of ClinicalChemistry, Collaborative, derivation of reference intervals for major clinical laboratory tests in Japan. Ann Clin Biochem. 2016;53(Pt 3):347‐356. 26362325 10.1177/0004563215608875

[26] Miura N , Kitamura H , Kameko M . Evaluation of the reference range of retinol‐binding protein (RBP) levels by the latex turbidimetric immunoassay. Jpn J Clin Pathol. 2009;57:195‐199. 19363988

[27] Watanabe N , Fujita T , Syono K , et al. Comparative study of various serum albumin measuring reagents. J Clin Lab Inst and Reagents. 2005;58:53‐58.

[28] Ichihara K . International trend on setting reference range. J Analyt Bio‐Sci. 2011;34:211‐217.

[29] Kinoshita S , Toyofuku M , Iida H , et al. Standardization of laboratory data and establishment of reference intervals in the Fukuoka Prefecture. Clin Chem Lab Med. 2001;39(3):256‐262. 11350024 10.1515/CCLM.2001.040

[30] ISO 11843‐1: Capability of detection –Part1: Terms and definitions. Genova 1997.

[31] Ichihara K , Boyd JC . IFCC Committee on Reference Intervals and Decision Limits (C‐RIDL), An appraisal of statistical procedures used in derivation of reference intervals. Clin Chem Lab Med. 2010;48(11):1537‐1551. 21062226 10.1515/CCLM.2010.319

[32] Marcovina SM , Gaur VP , Albers JJ . Biological variation of cholesterol, triglyceride, low‐ and high‐ density lipoprotein cholesterol, Lipoprotein(a), and apolipoproteins A‐ and B. Clin Chem. 1994;40(4):574‐578. 8149613

[33] Ichihara K , Ozarda Y , Barth JH , et al. A global multicenter study on reference values: 1. Assessment of methods for derivation and comparison of reference intervals. Clin Chim Acta. 2017;467:70‐82. 27666761 10.1016/j.cca.2016.09.016

[34] Japan Clinical Sanitation Association Technical Association . Precision and accuracy assessment method for quantitative test in clinical chemistry guidelines. Jpn Lab Std Council J. 1999;14:3‐25.

[35] Petersen PH , Fraser CG , Lund F , et al. Confirmation of analytical performance characteristics required for the reference change value applied in patient monitoring. Scand J Clin Lab Invest. 2015;75(7):628‐630. 26305425 10.3109/00365513.2015.1057897

[36] Hosogaya S , Ozaki Y . Domestic and international trends concerning allowable limits of error in external quality assessment scheme. Jpn J Clin. Pathol. 2005;53:547‐553. 16026083

[37] Miyake N . Reference value for metabolic syndrome checkup and some problems Concept of individual reference range. Jpn J Clin Pathol. 2009;57:217‐233. 19860216

[38] Tonks DB . A study of accuracy and precision of clinical chemistry determinations in 170 Canadian Laboratories. Clin Chem. 1963;9:217‐233. 13985504

[39] Cotlove E , Harris EK , Williams GZ . Biological and analytic components of variation in long–term studies of serum constituents in normal subjects: Ⅲ. Physiological and medical implications. Clin Chem. 1970;16:1028‐1032. 5481563

[40] Fraser CG . The application of theoretical goals based upon biological variation in proficiency testing. Arch Pathol Lab Med. 1988;112(4):404‐415. 3355342

[41] Williams GZ . Individuality of clinical biochemical patterns in preventive health maintenance. J Occup Med. 1967;9:567‐570. 6054748

[42] Eugene EK . Effects of intra and interindividual variation on the appropriate use of normal ranges. Clin Chem. 1974;20:1535‐1542. 4430131

[43] Fraser CG . Biological variation: from principles to practice. Washington, DC: AACC Press; 2001. ISBN-10:1890883492.

