# Supplementary figures and images for: Comparative evaluation of methods to determine intra‐individual reference ranges in nutrition support team (NST)‐related tests
Source: J Clin Lab Anal. 2020 Oct 27;35(2):e23639. doi: 10.1002/jcla.23639 (PMC7891514; doi:10.1002/jcla.23639)

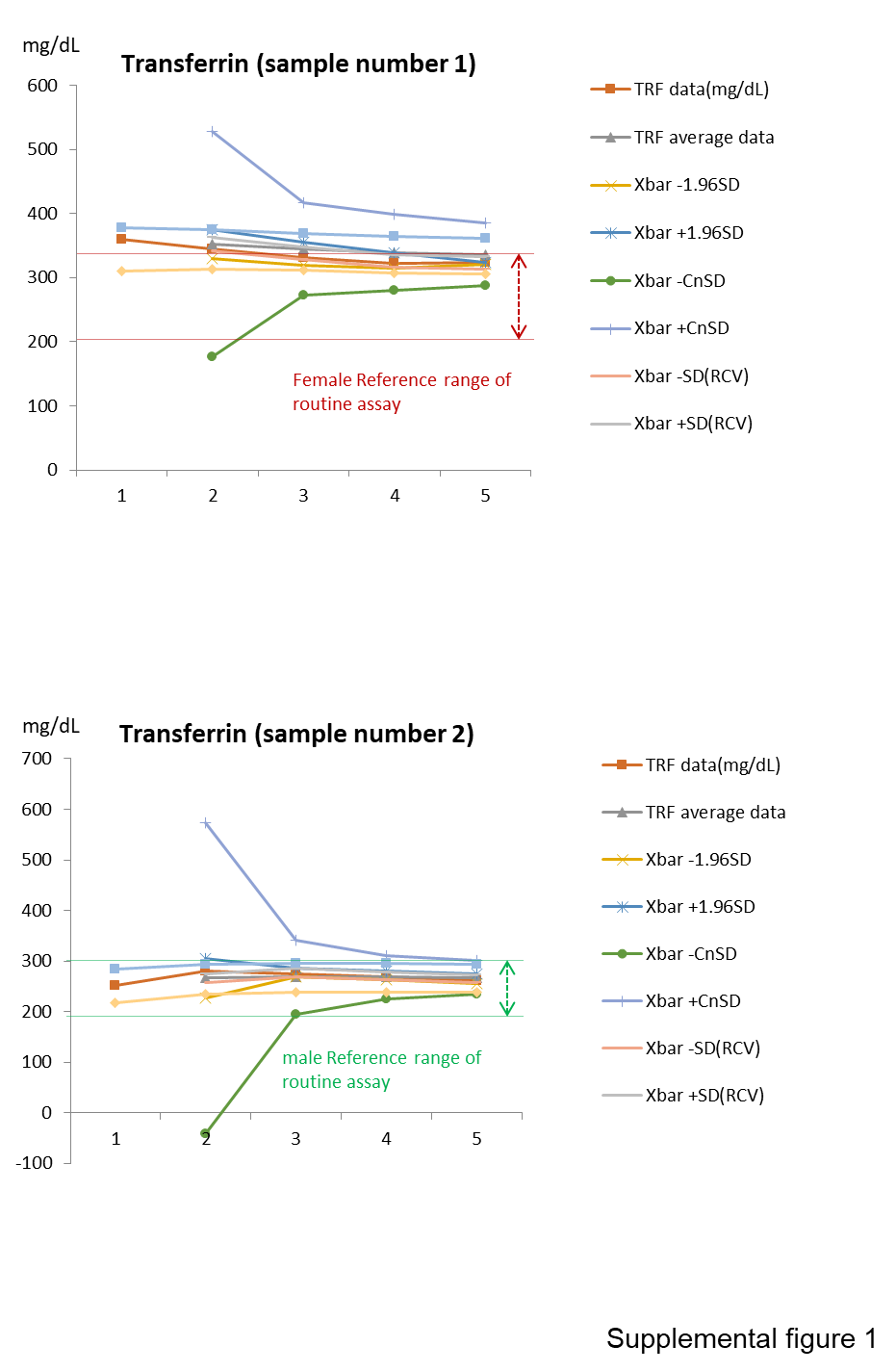


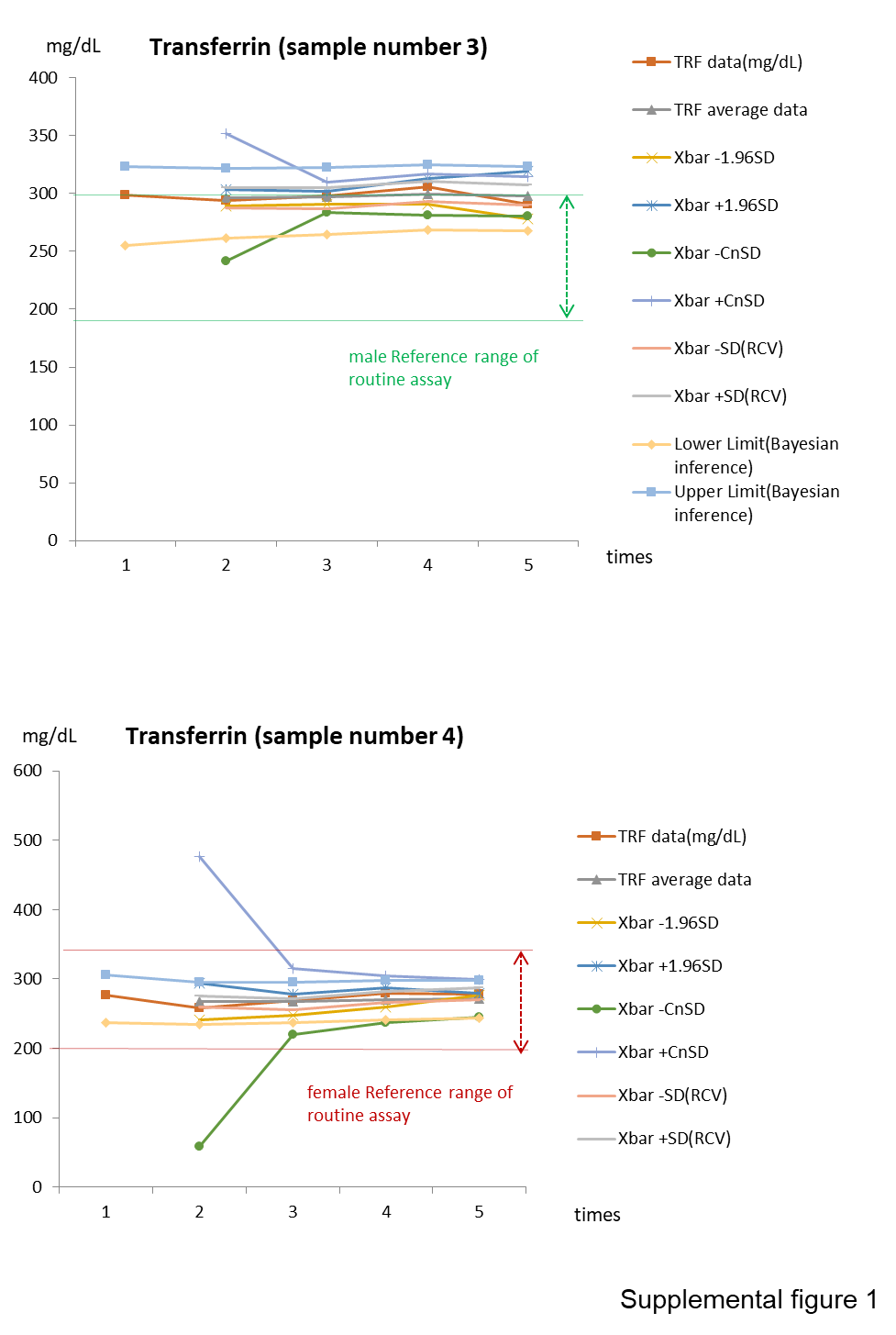


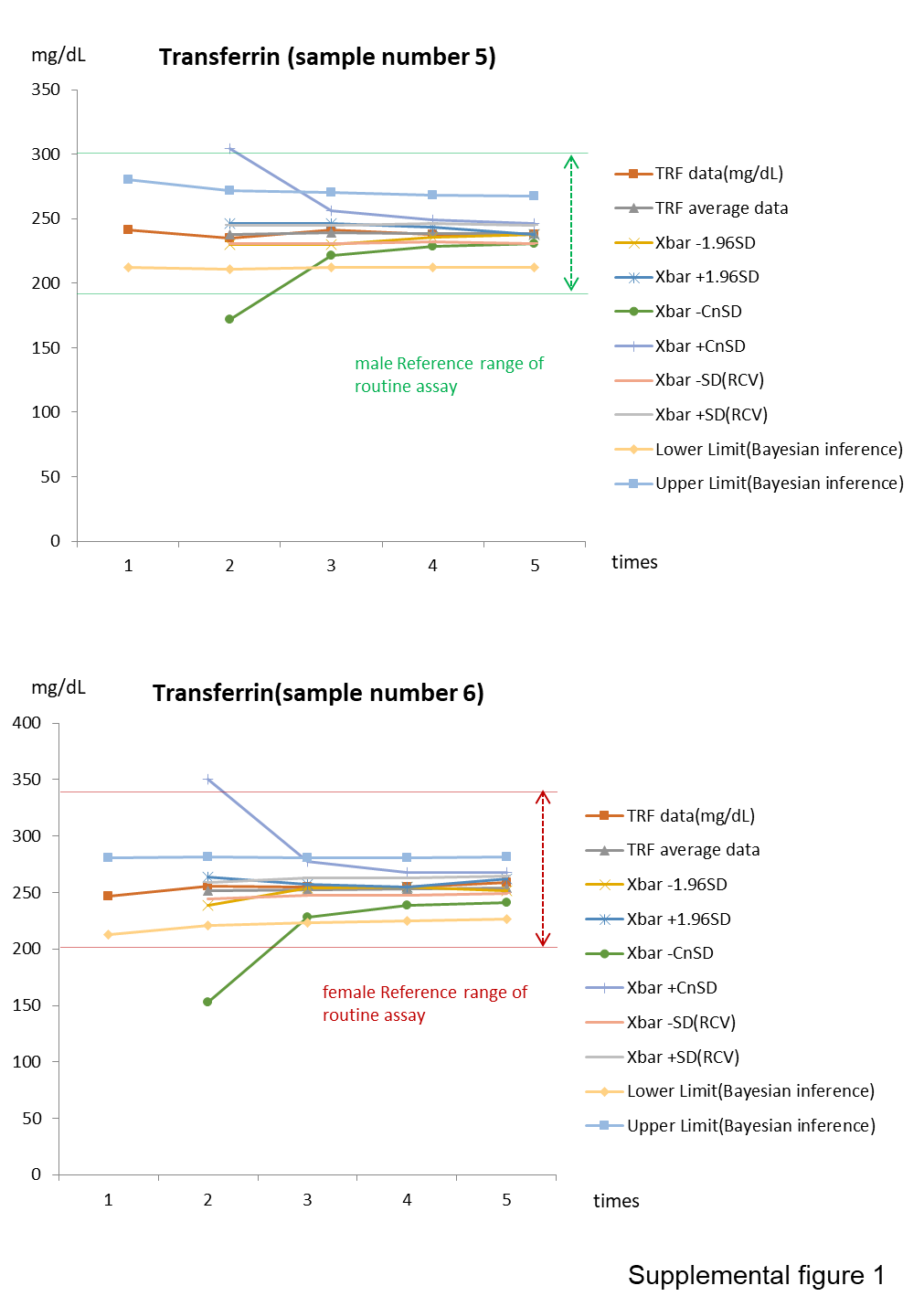


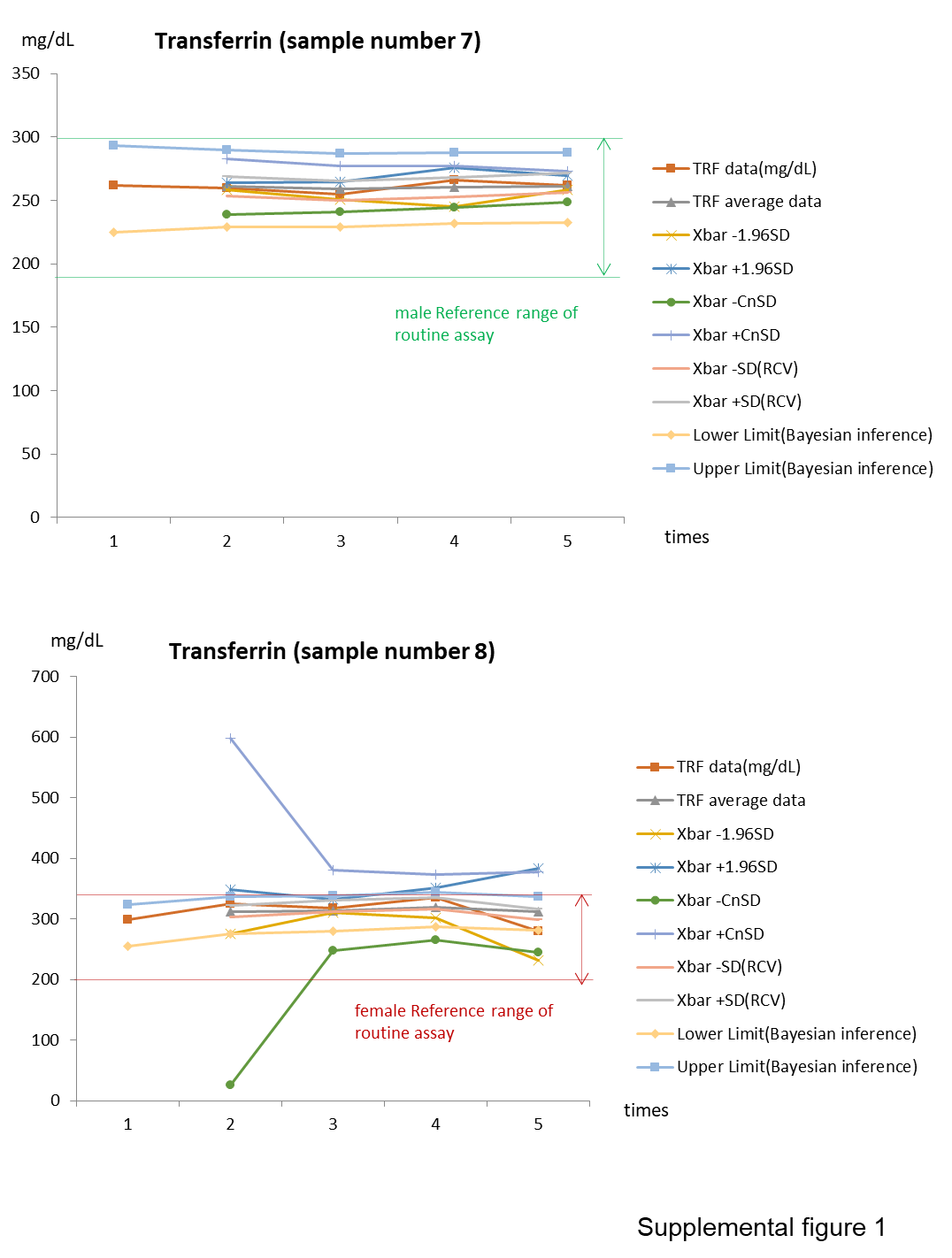


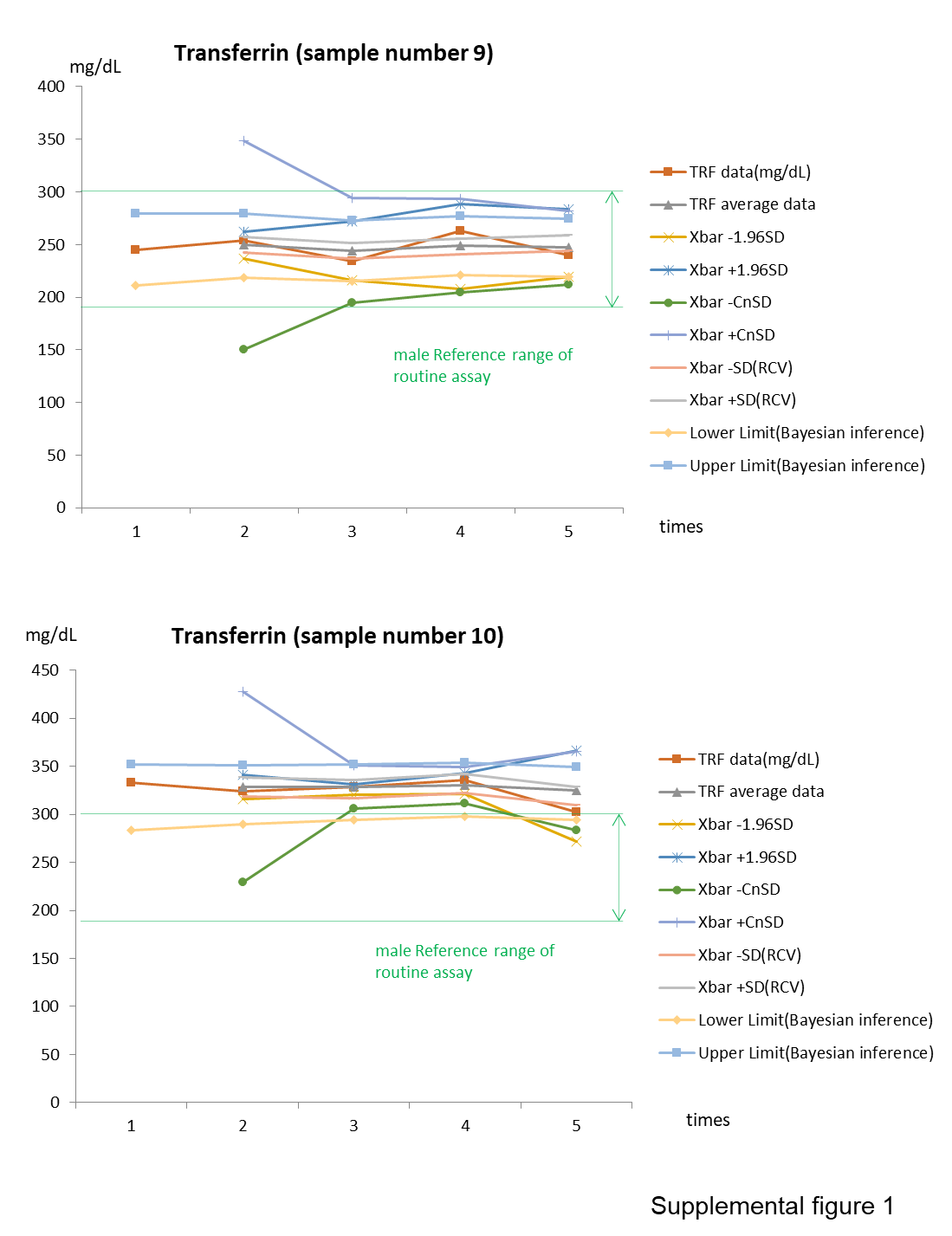


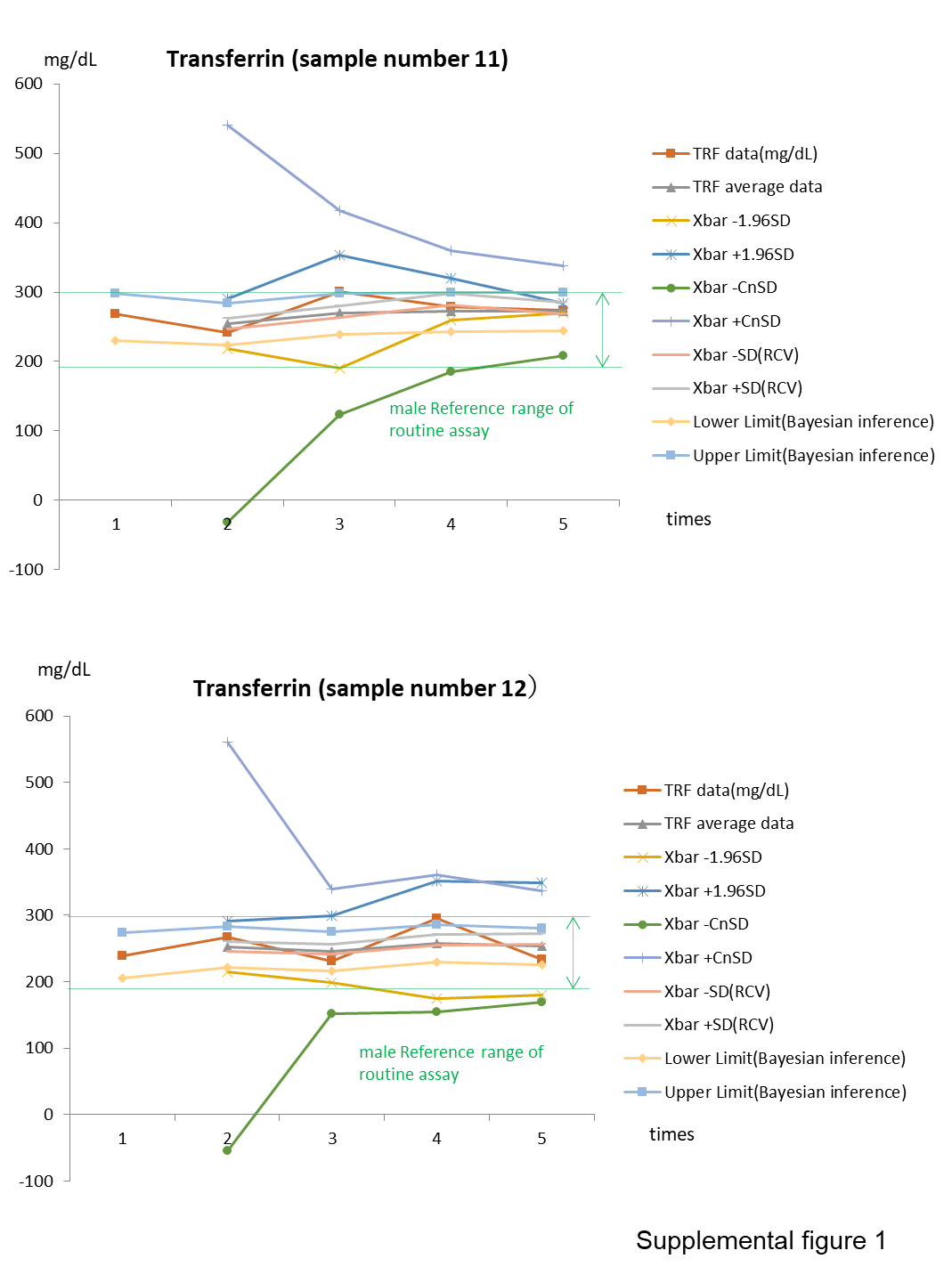


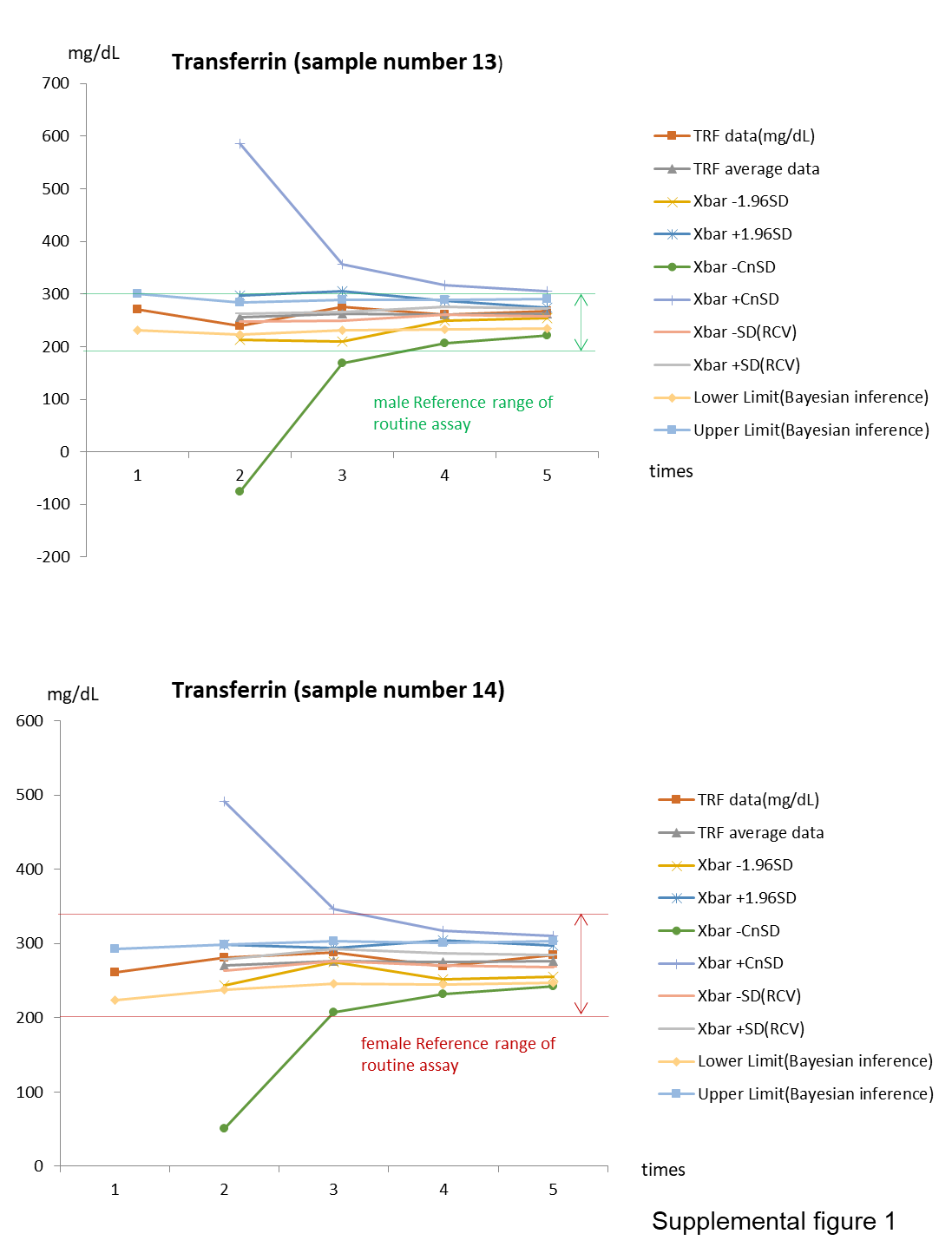


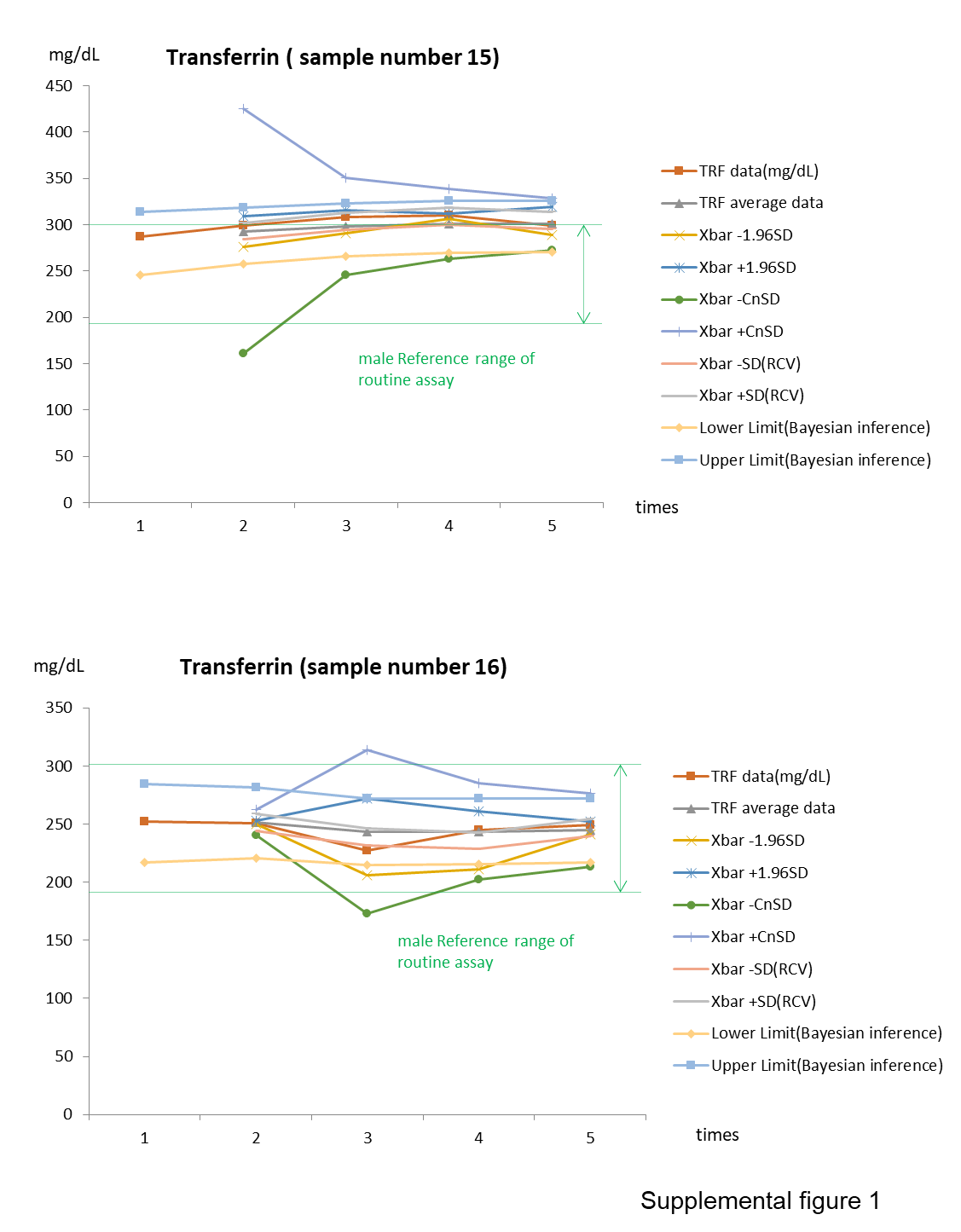


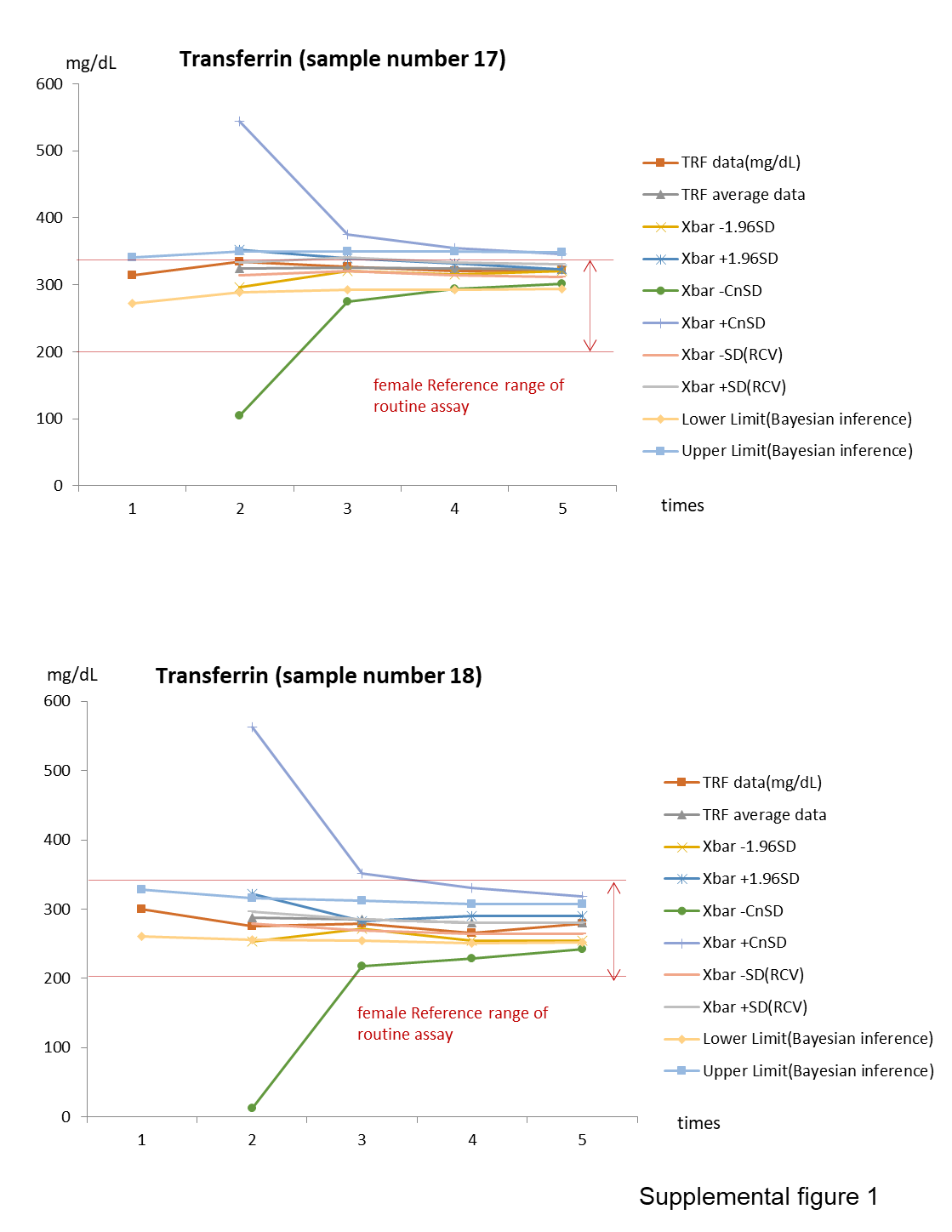


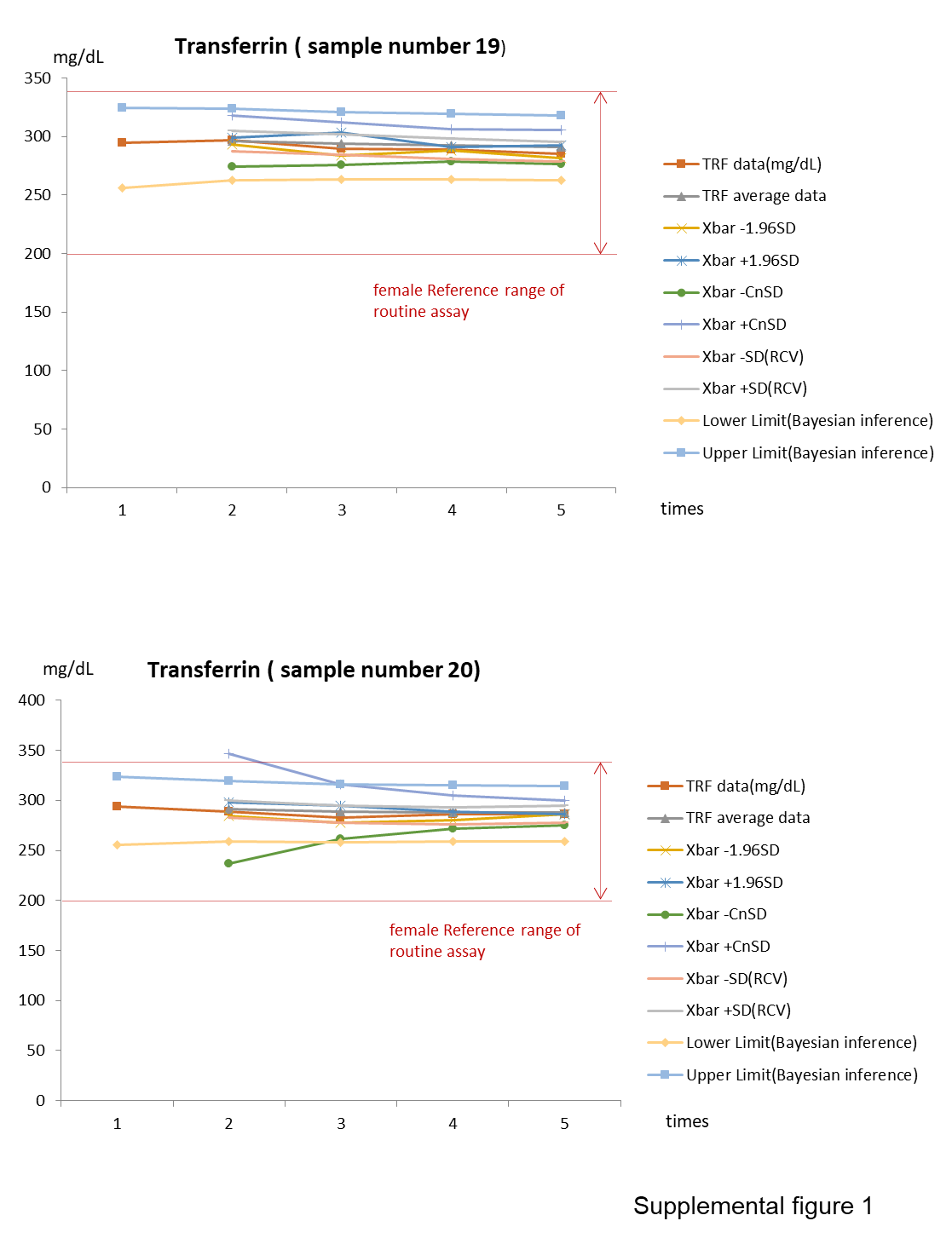


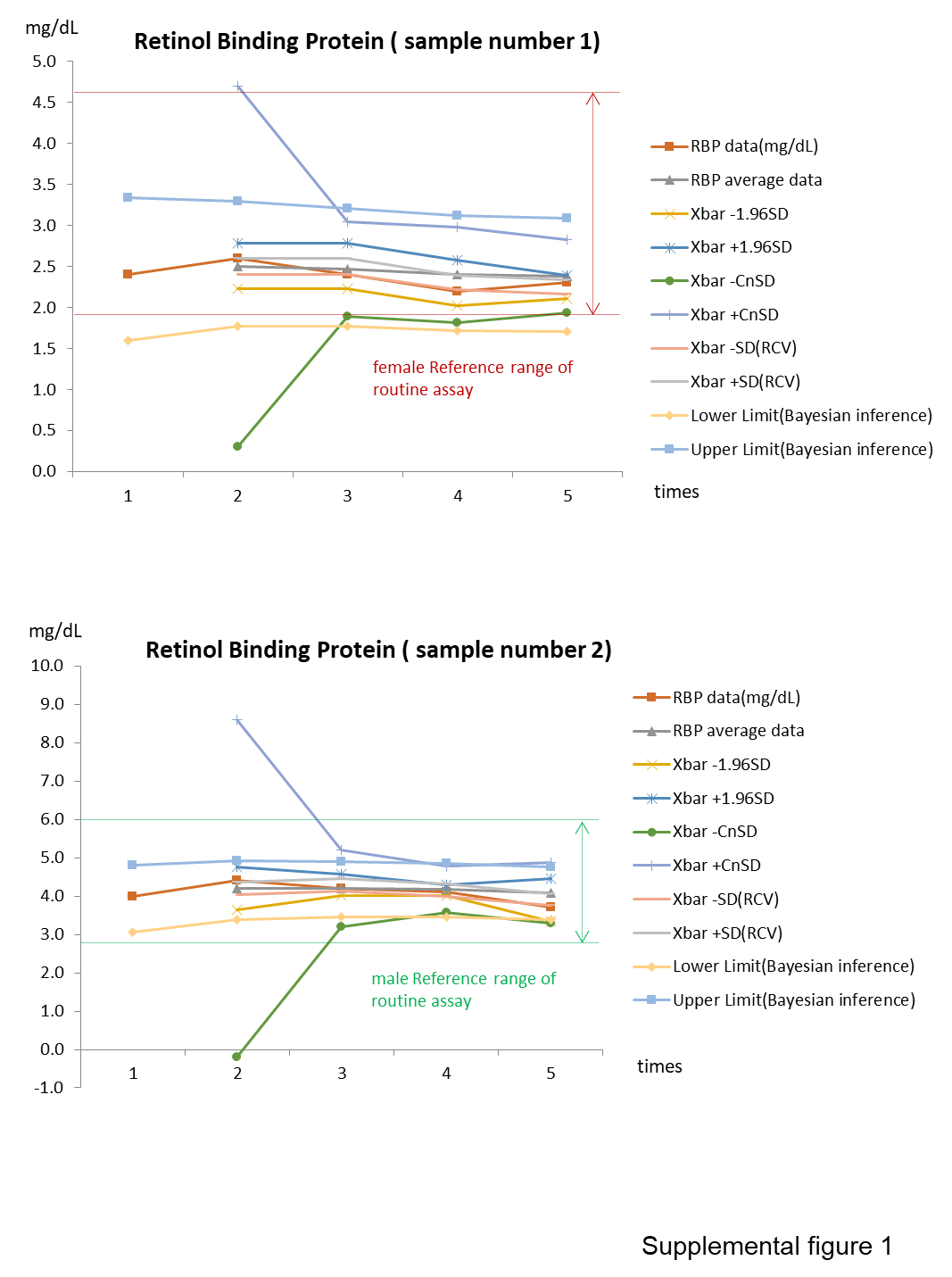


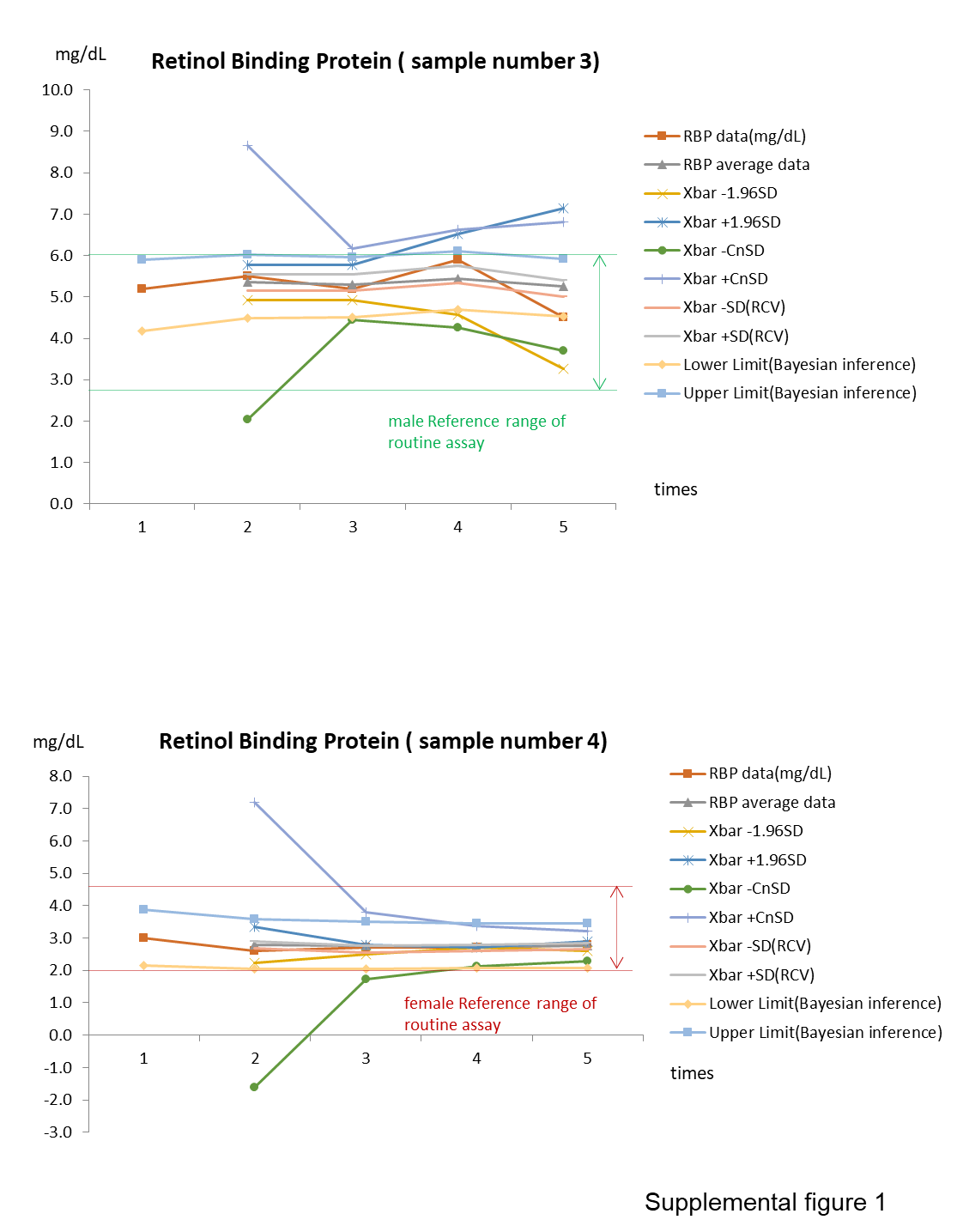


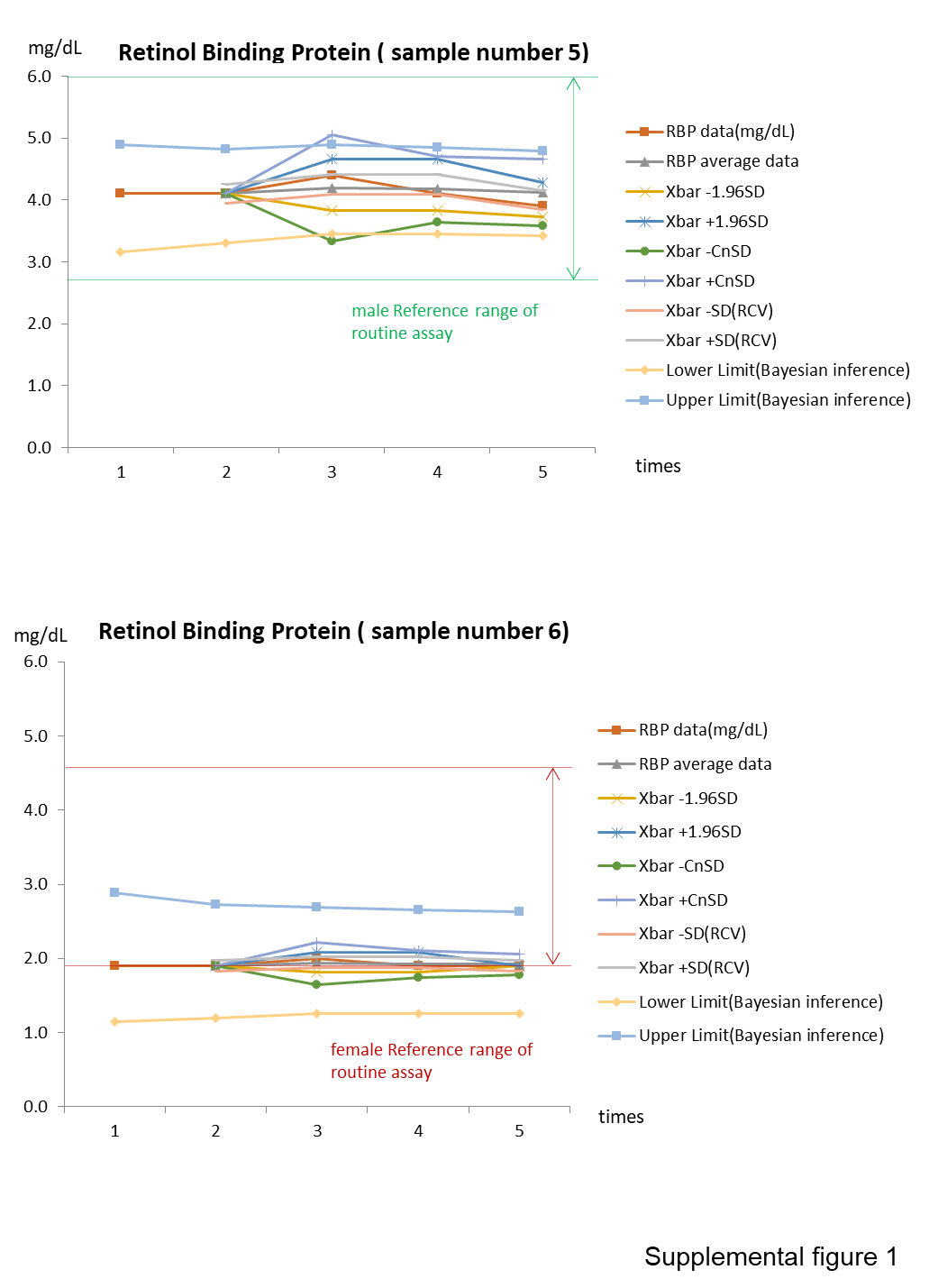


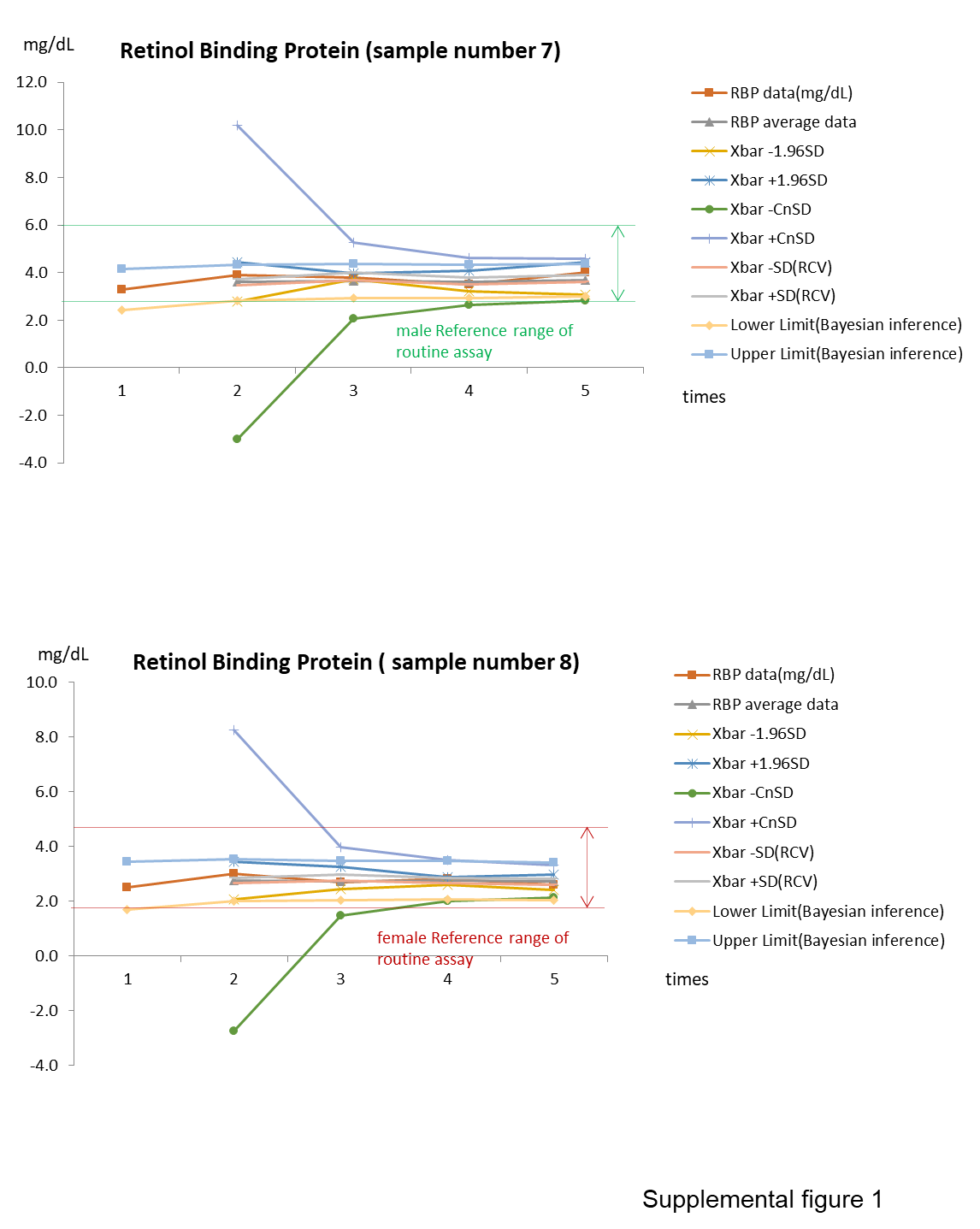


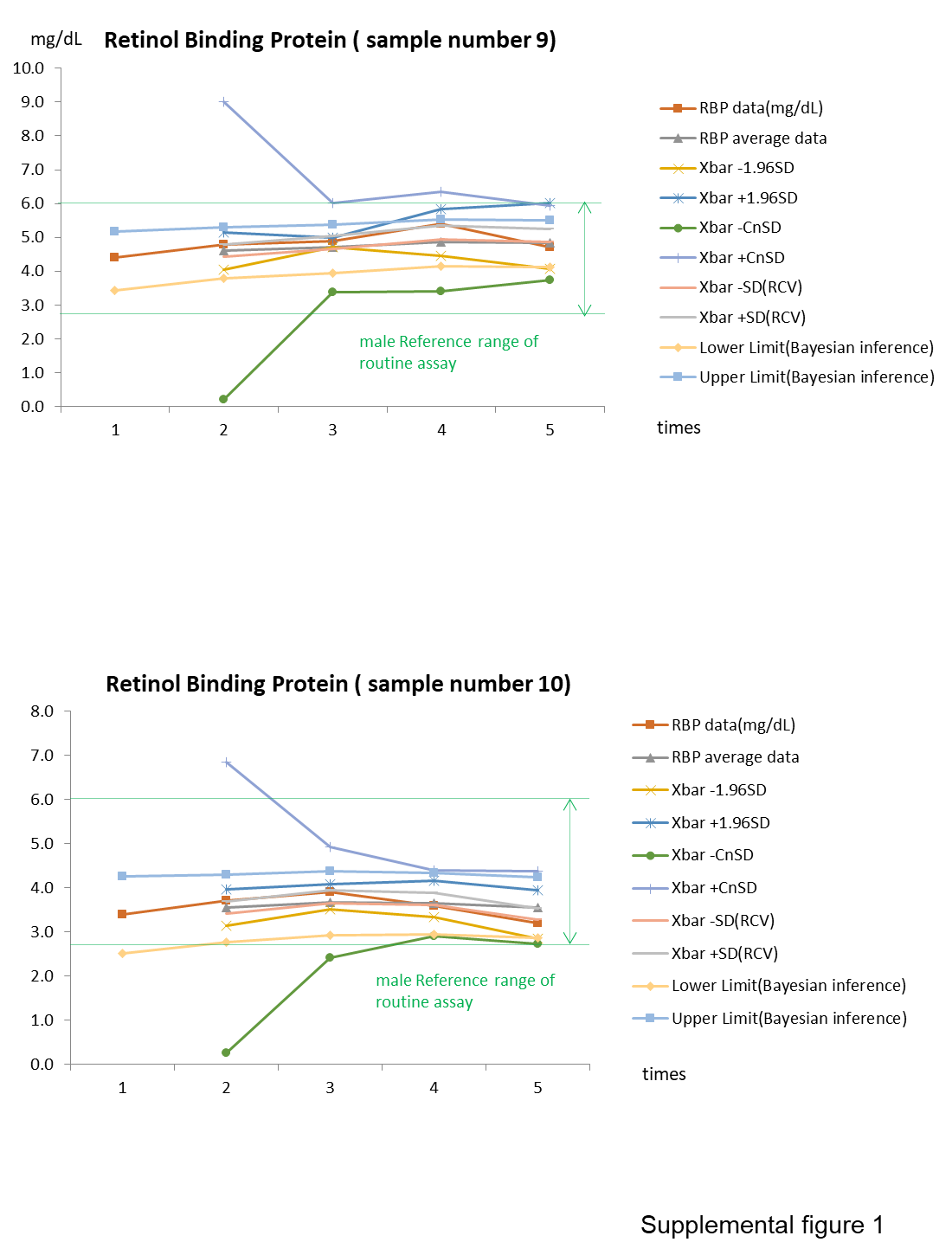


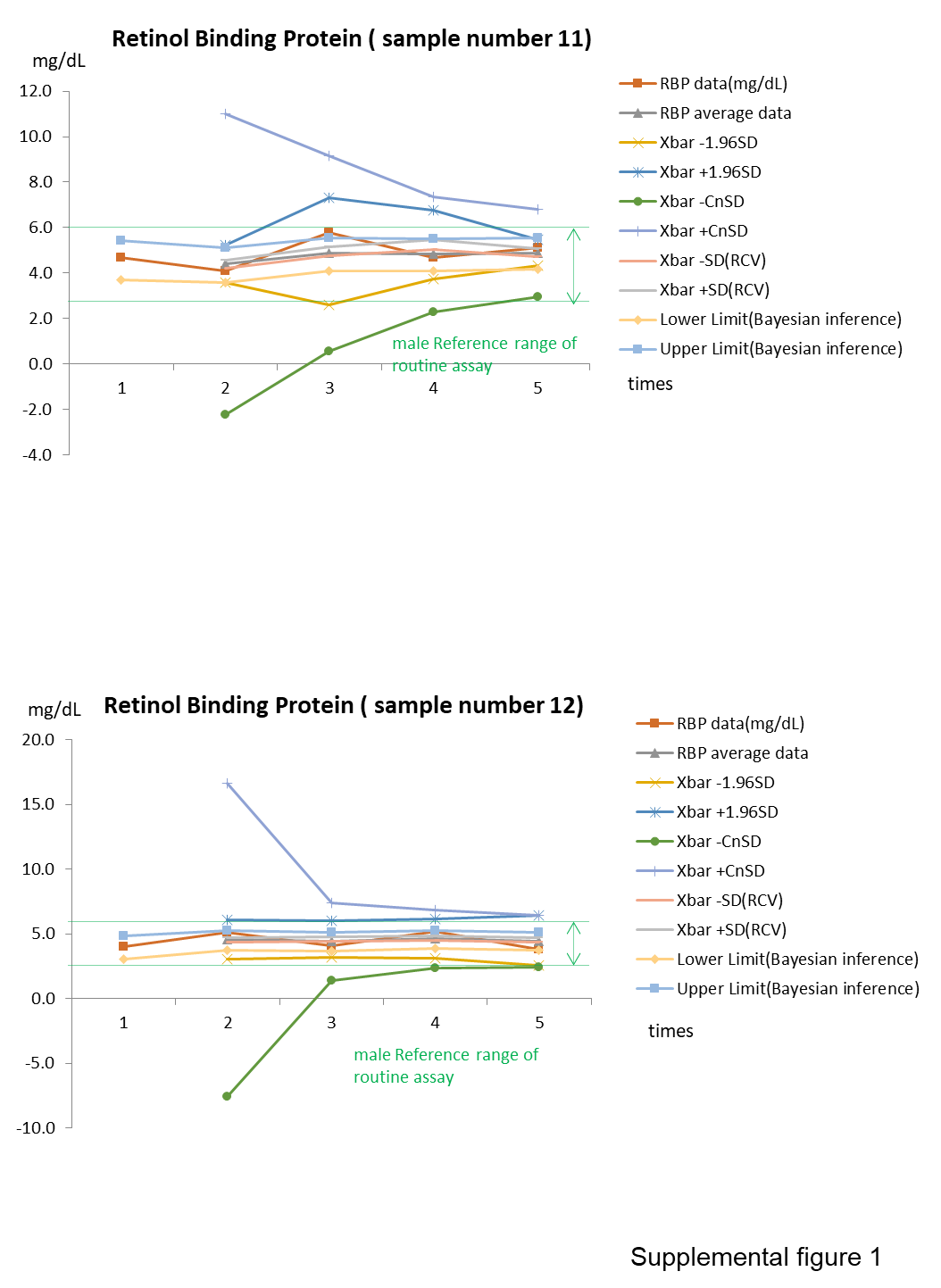


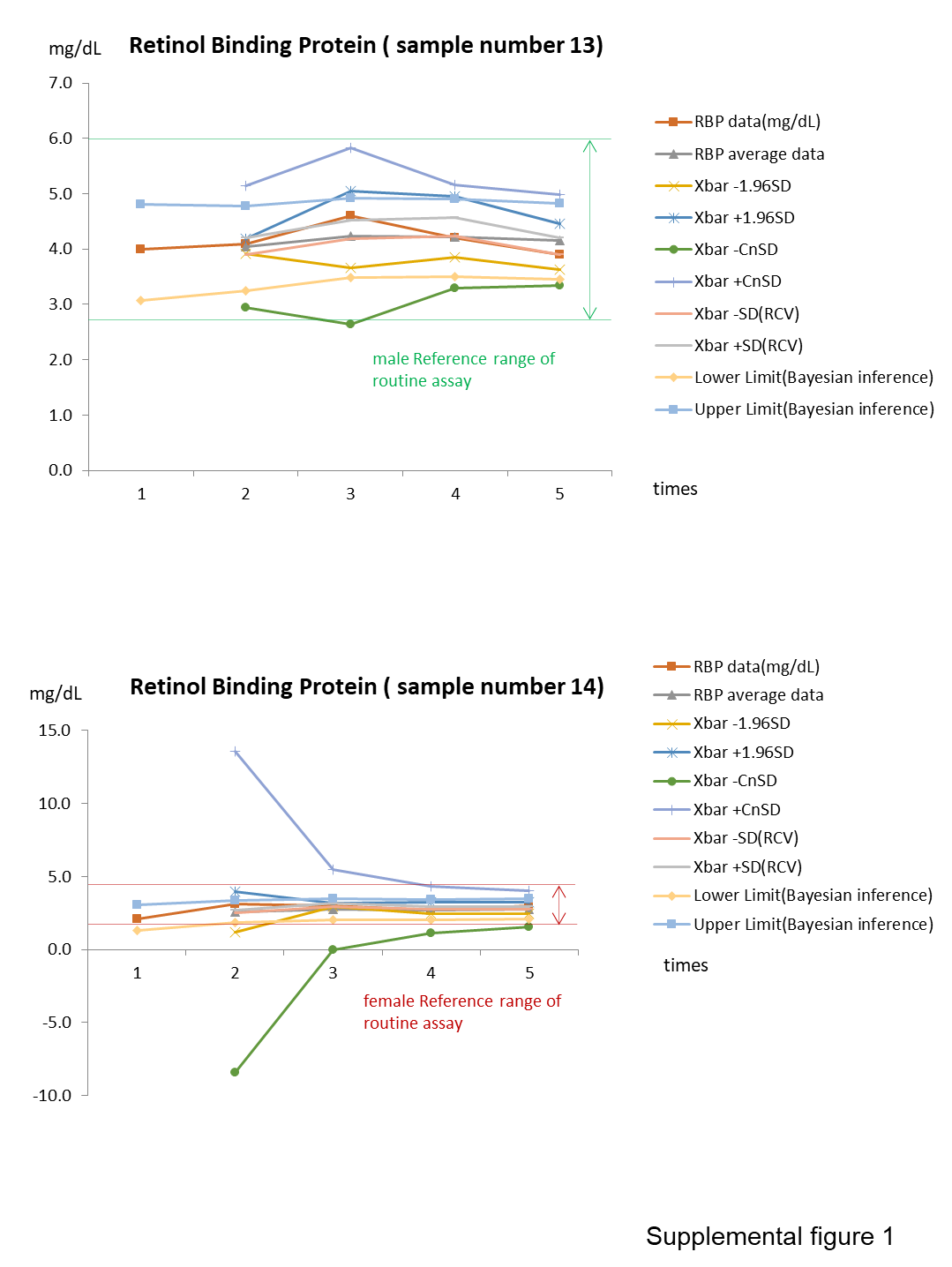


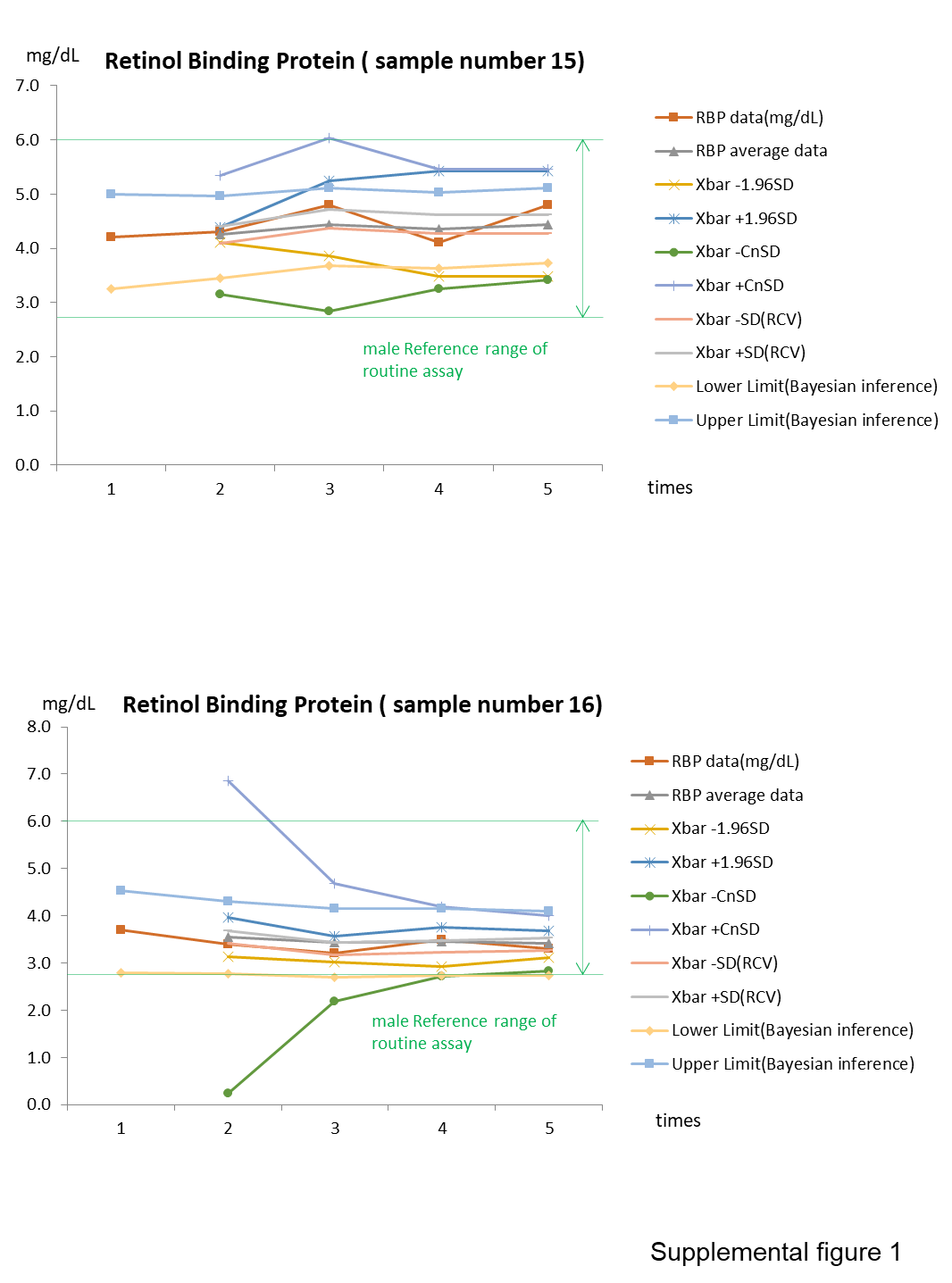


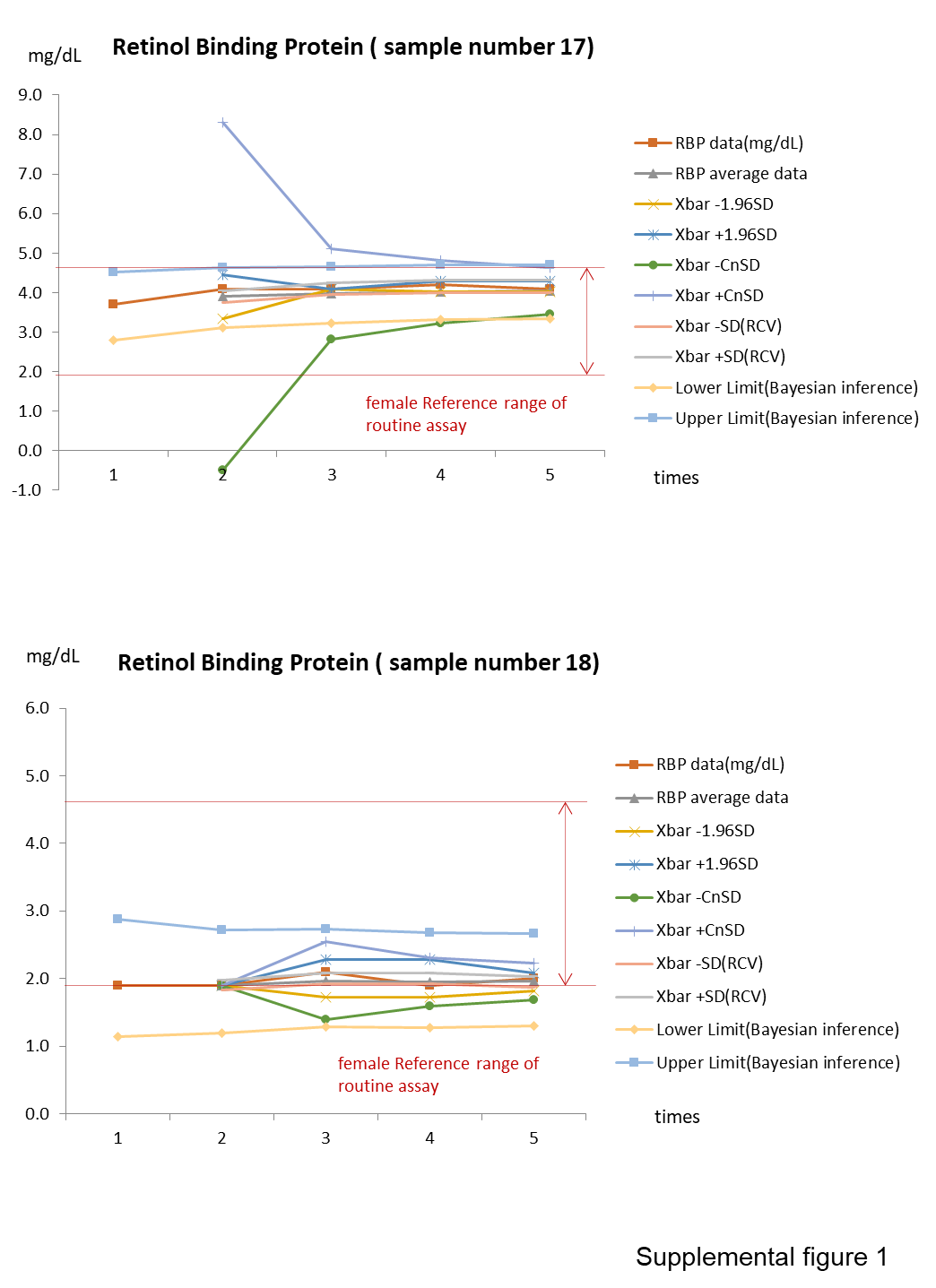


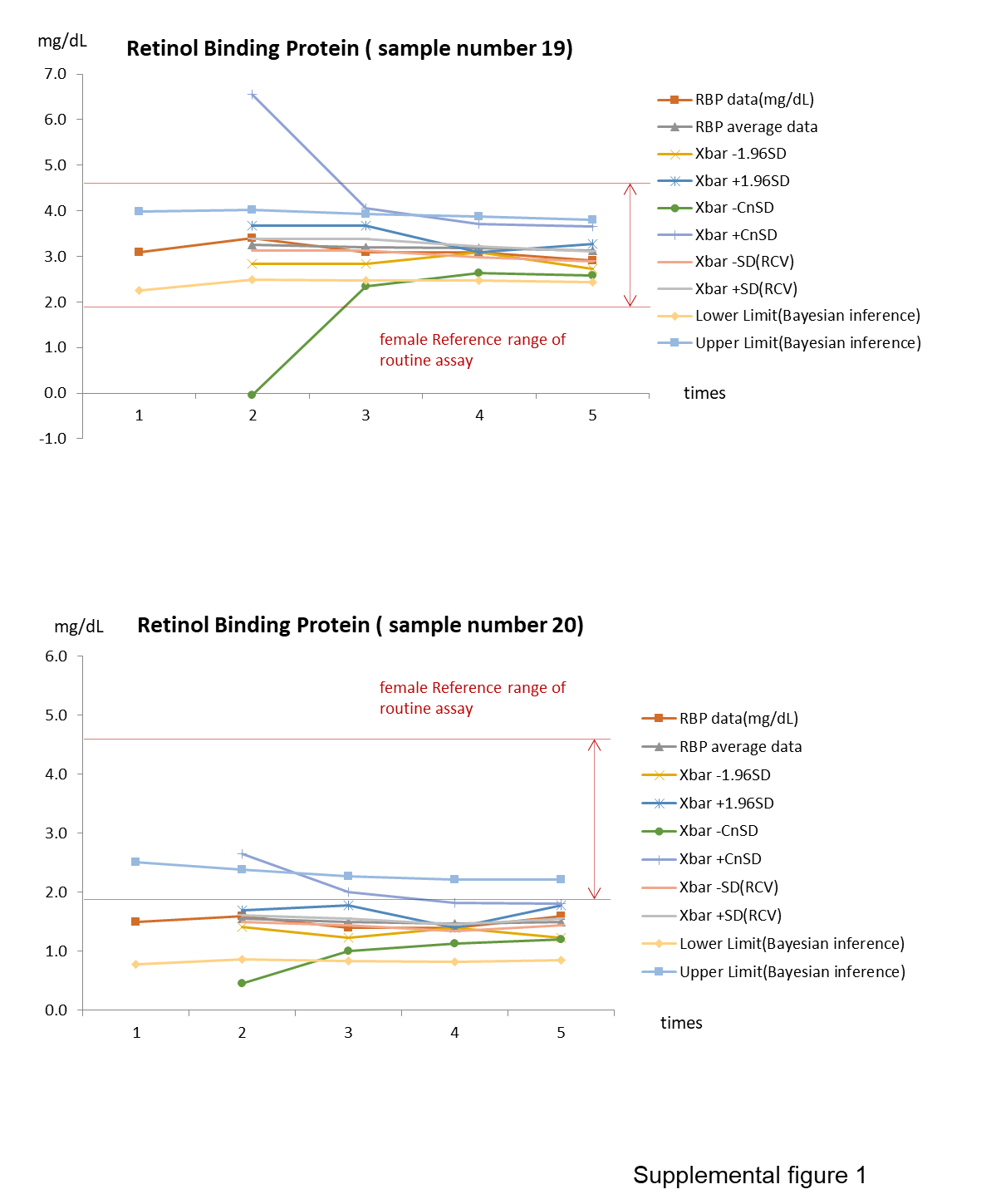


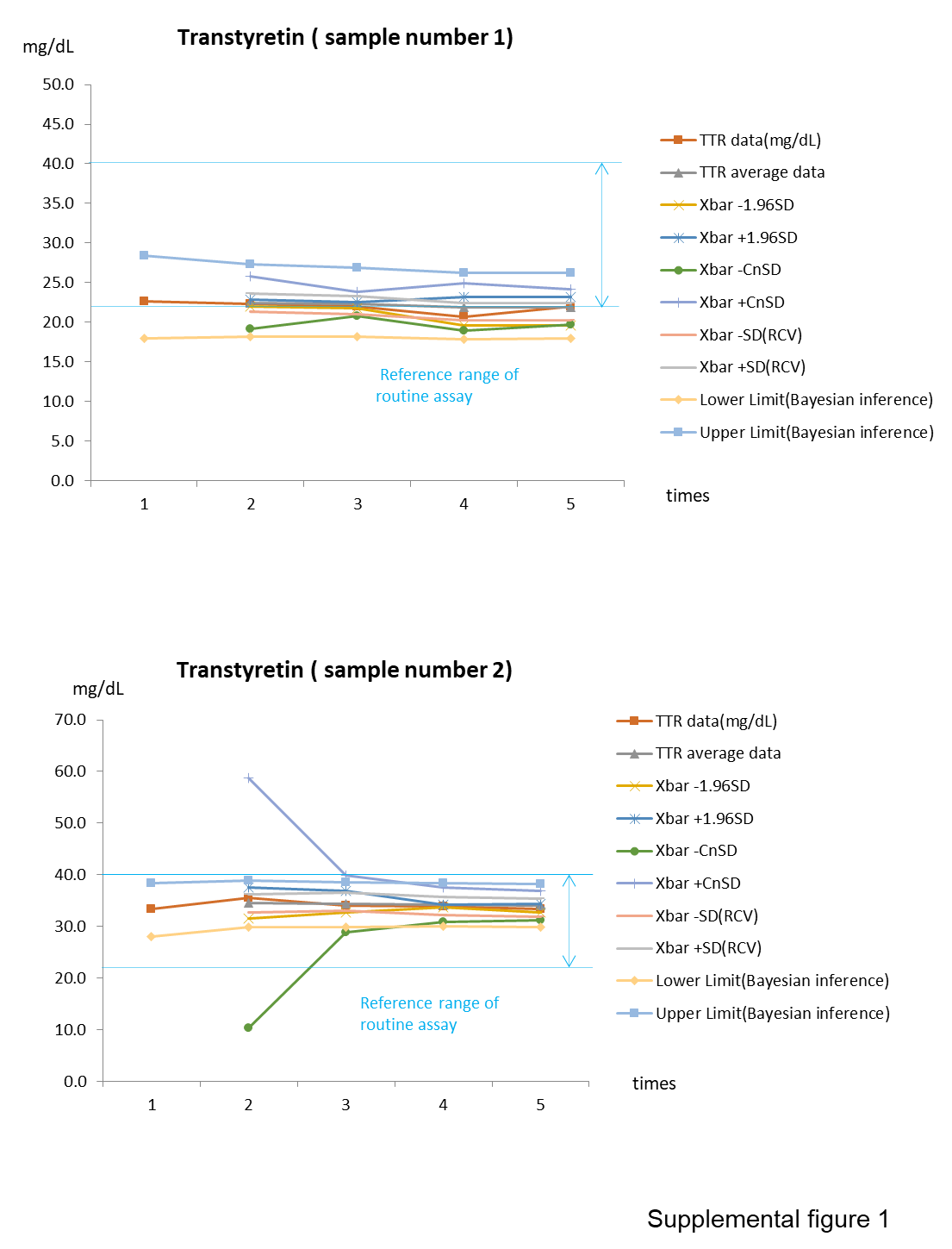


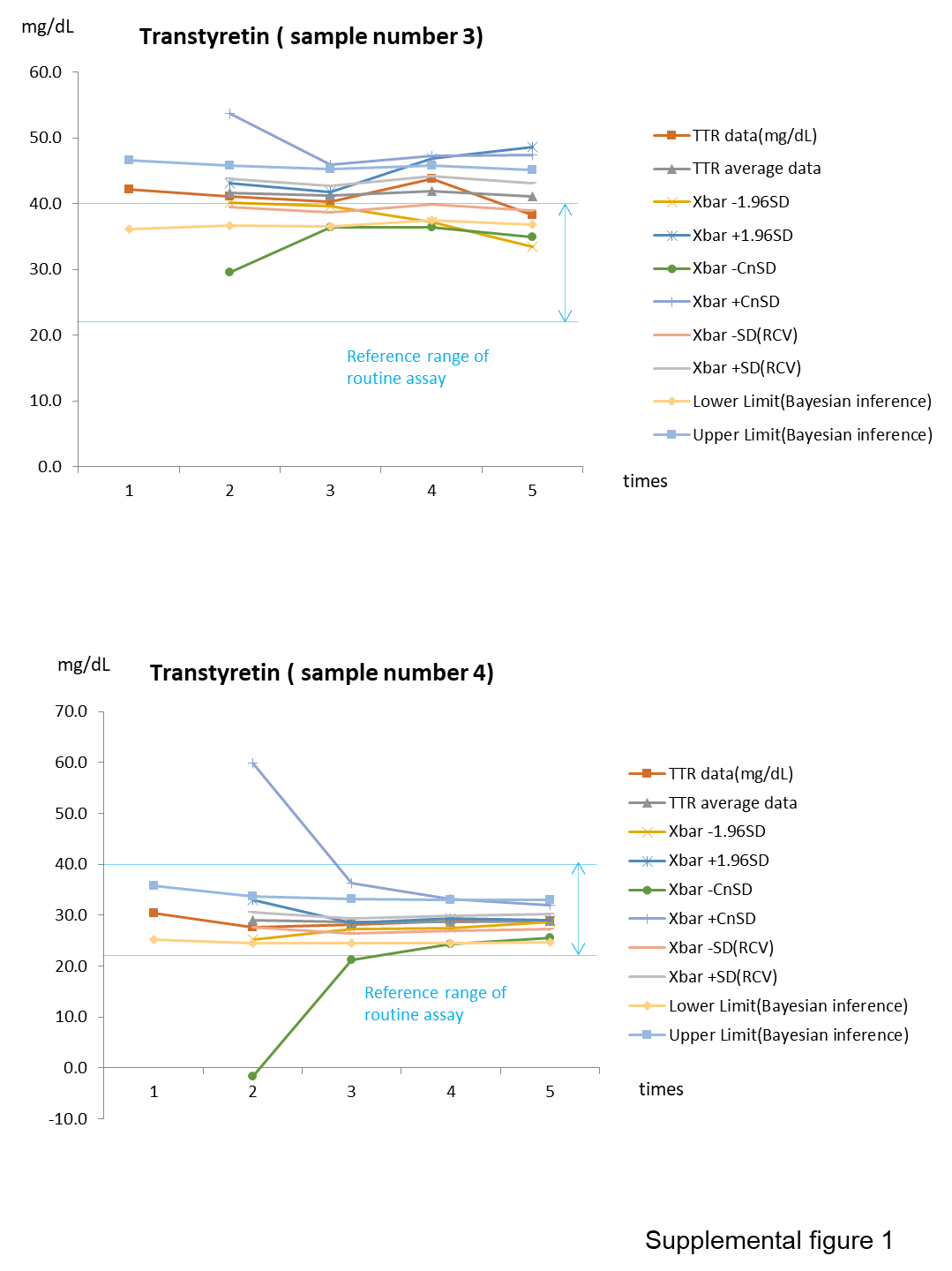


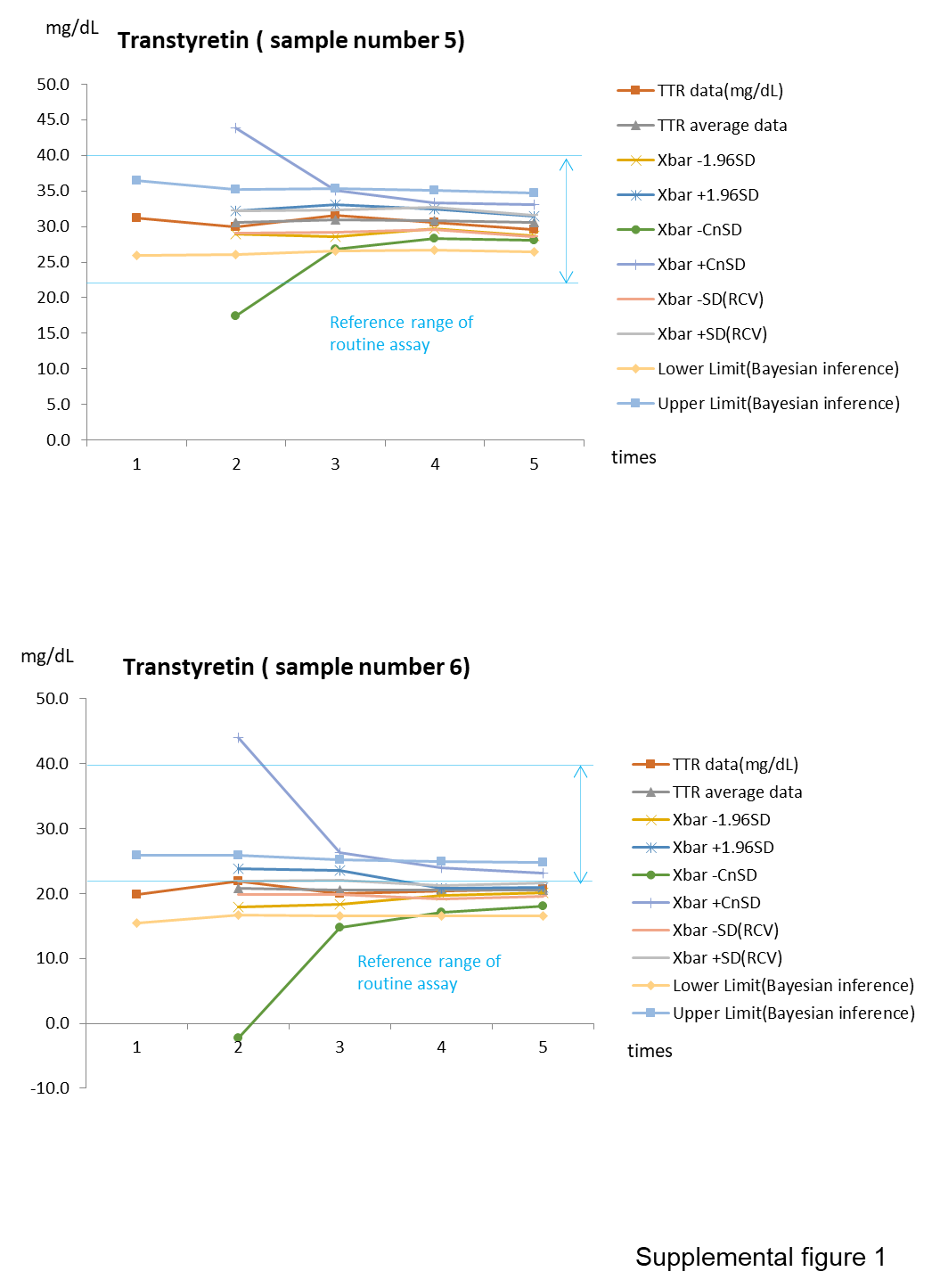


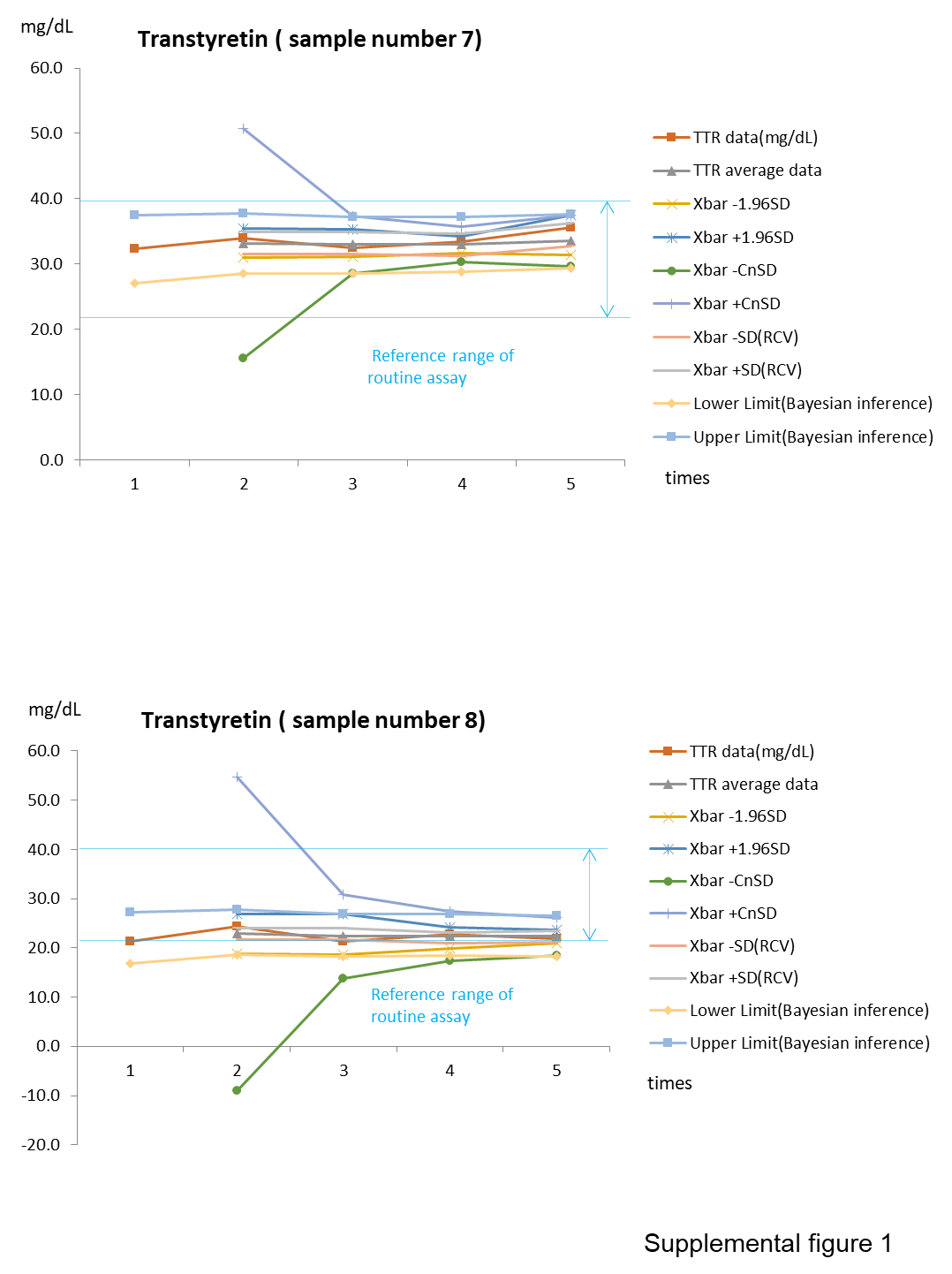


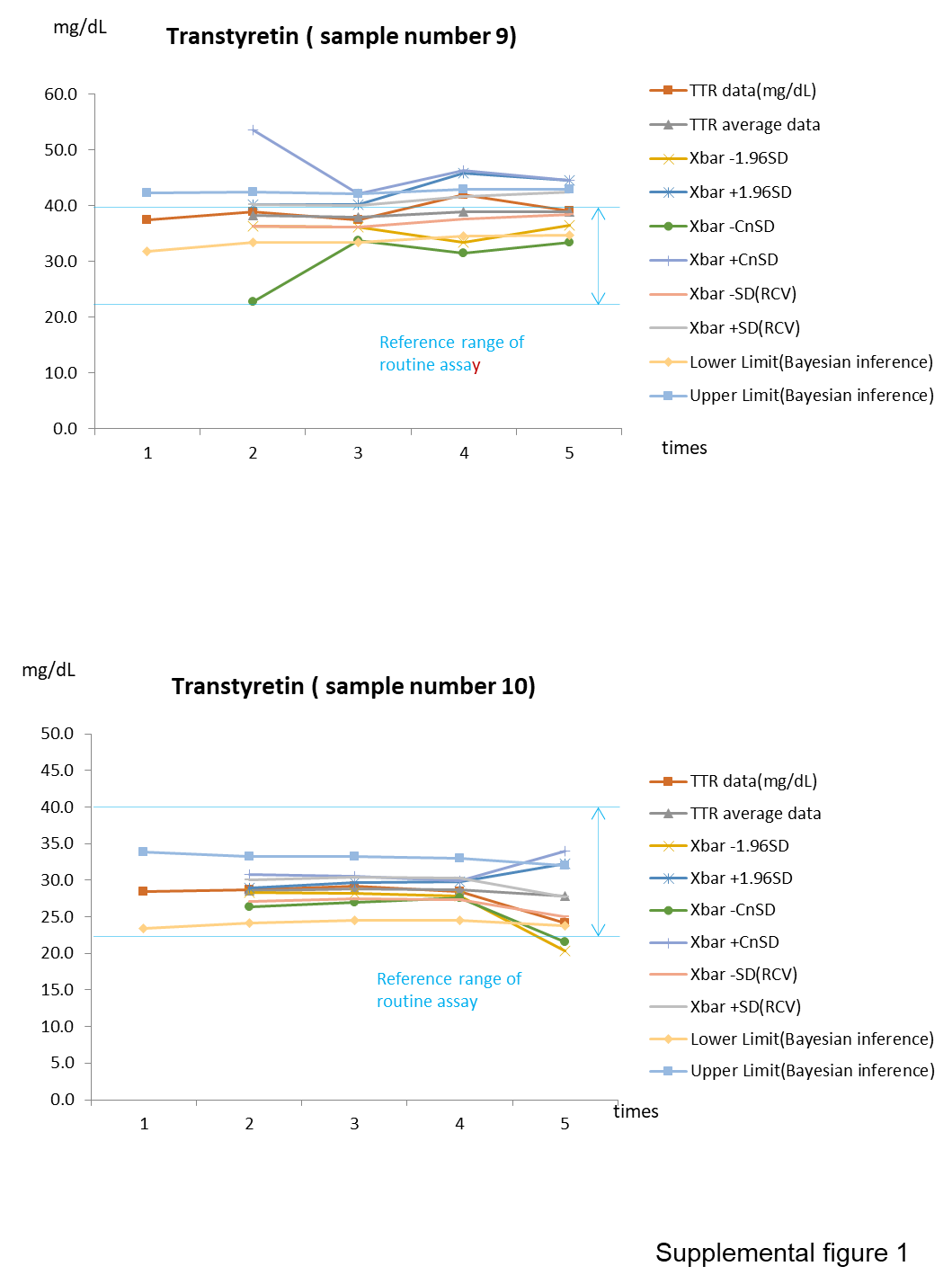


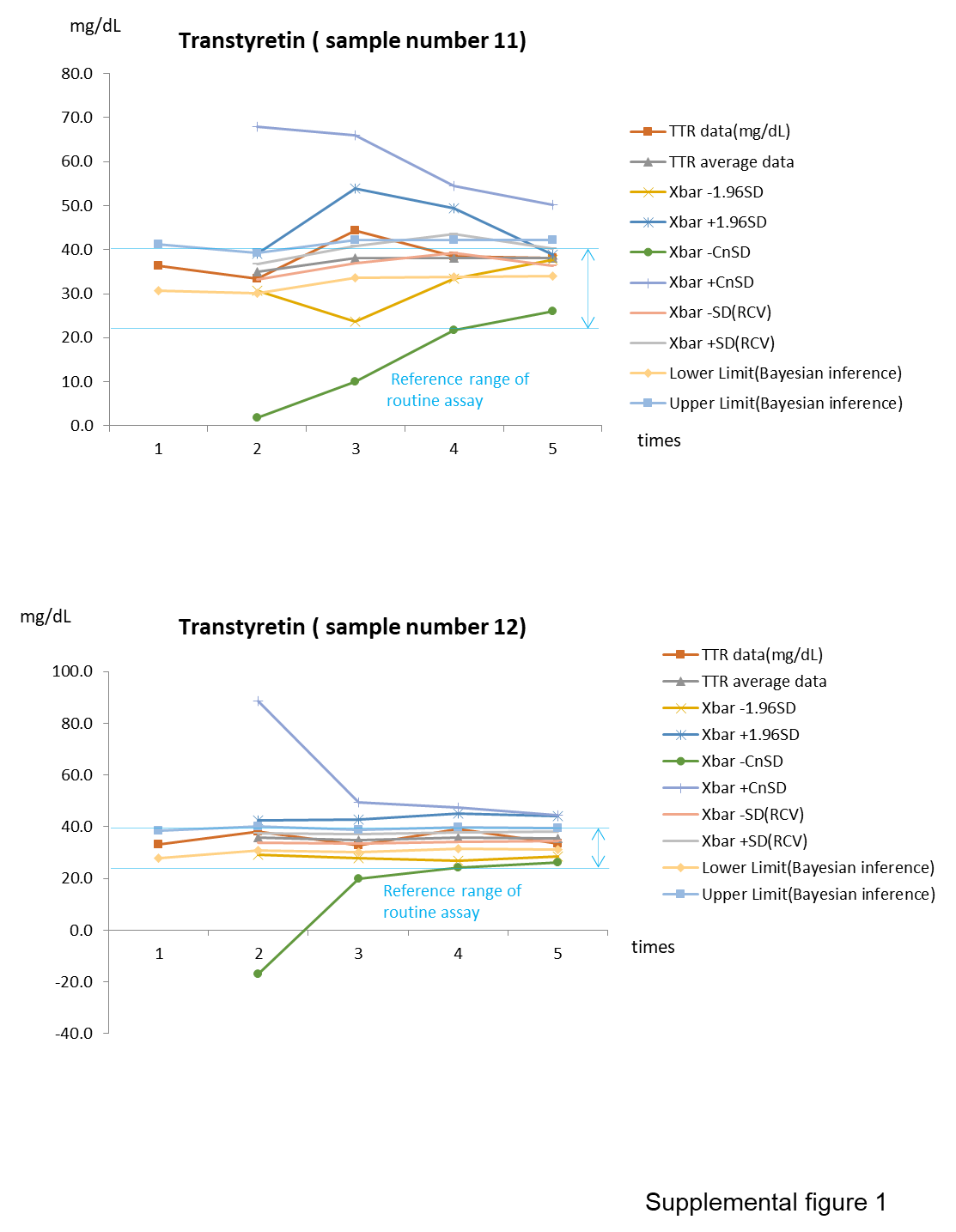


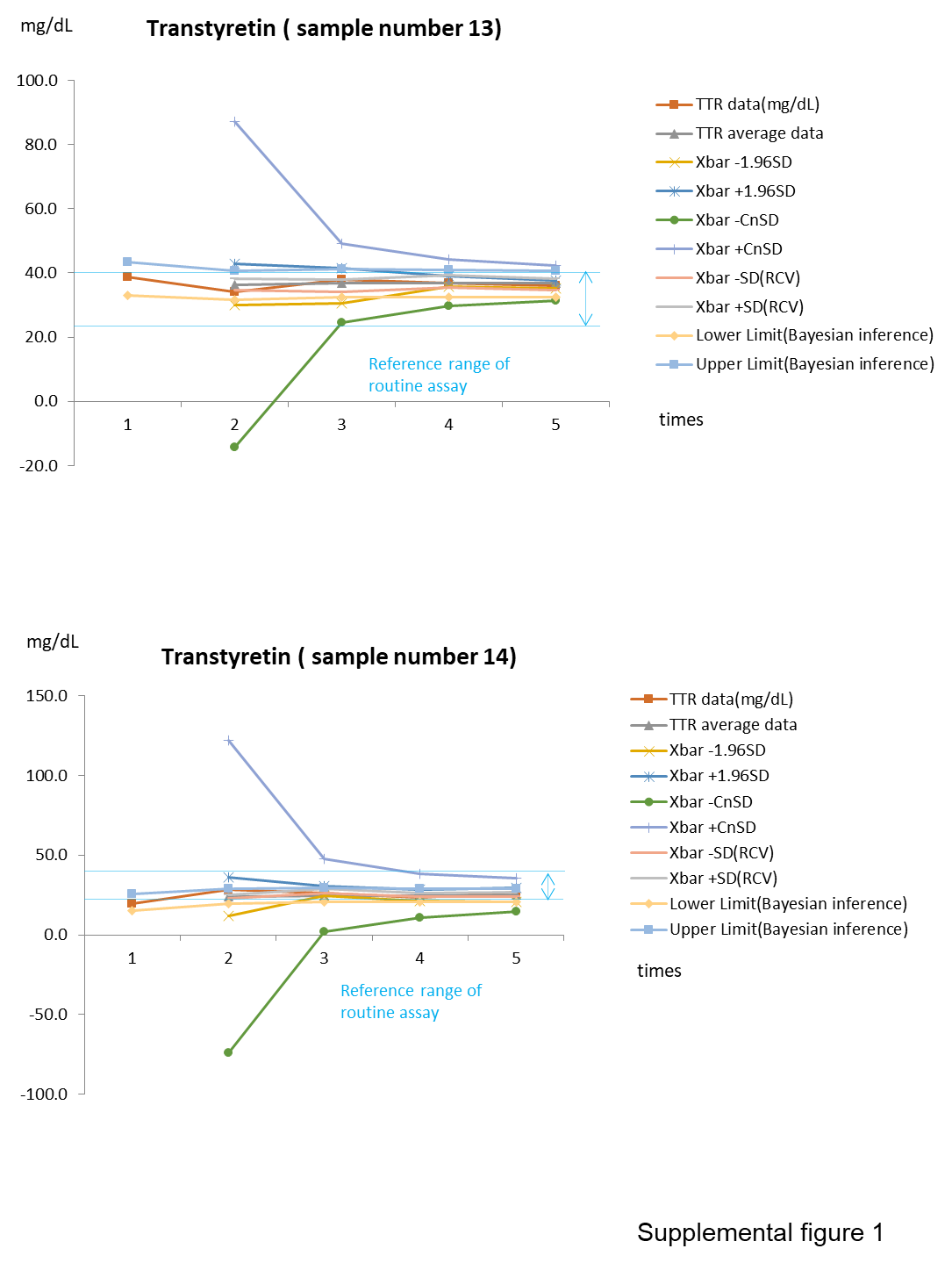


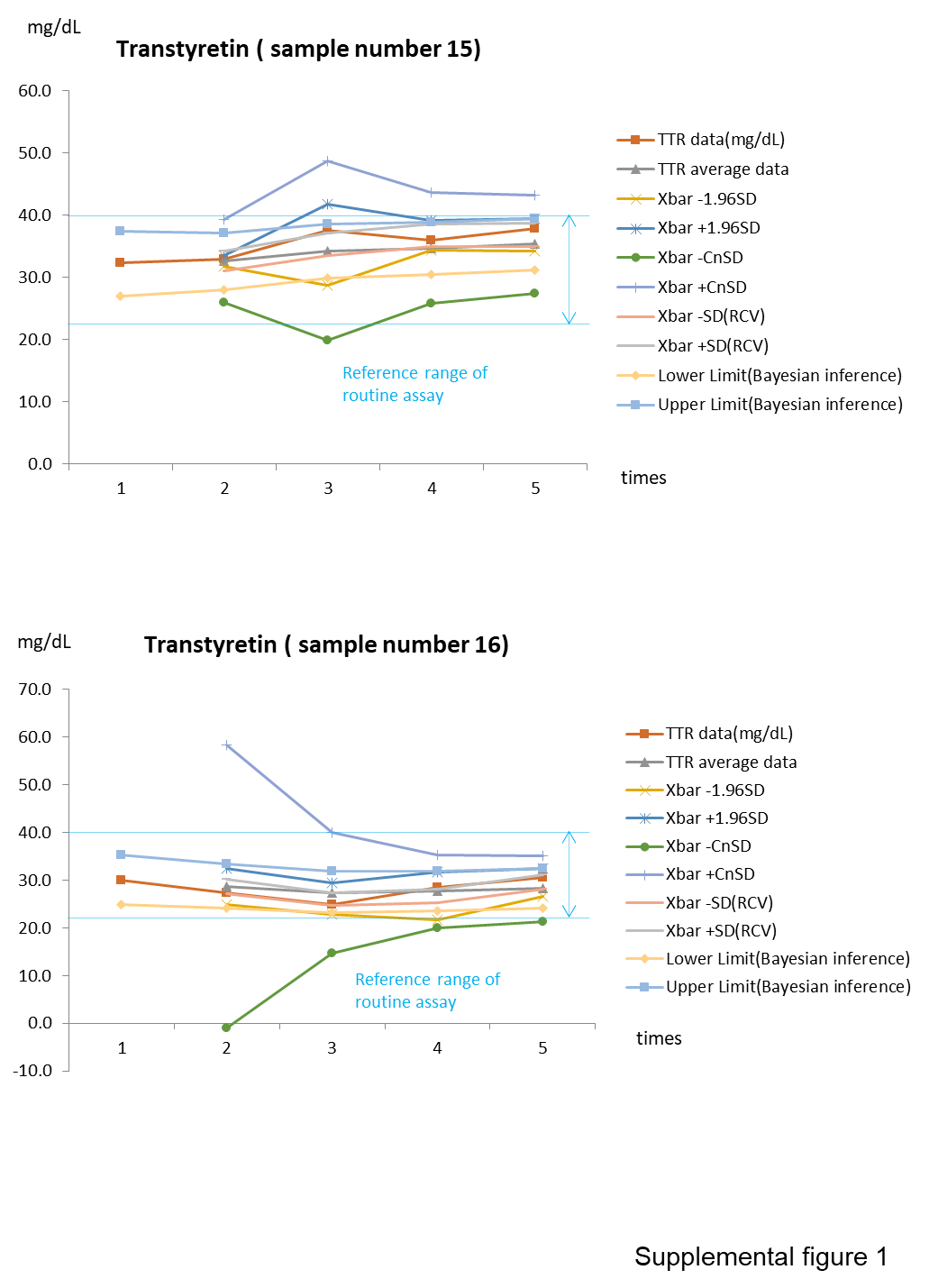


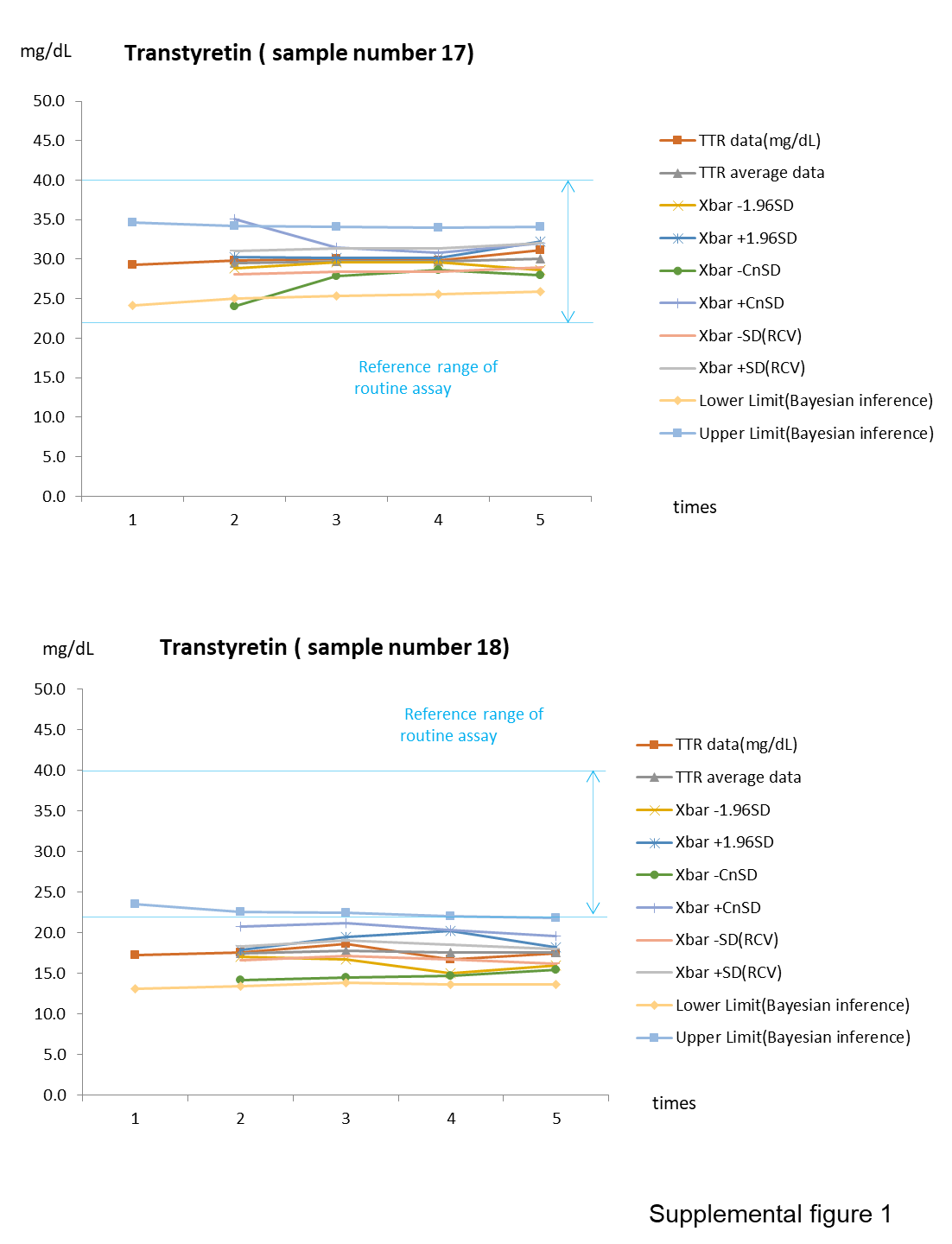


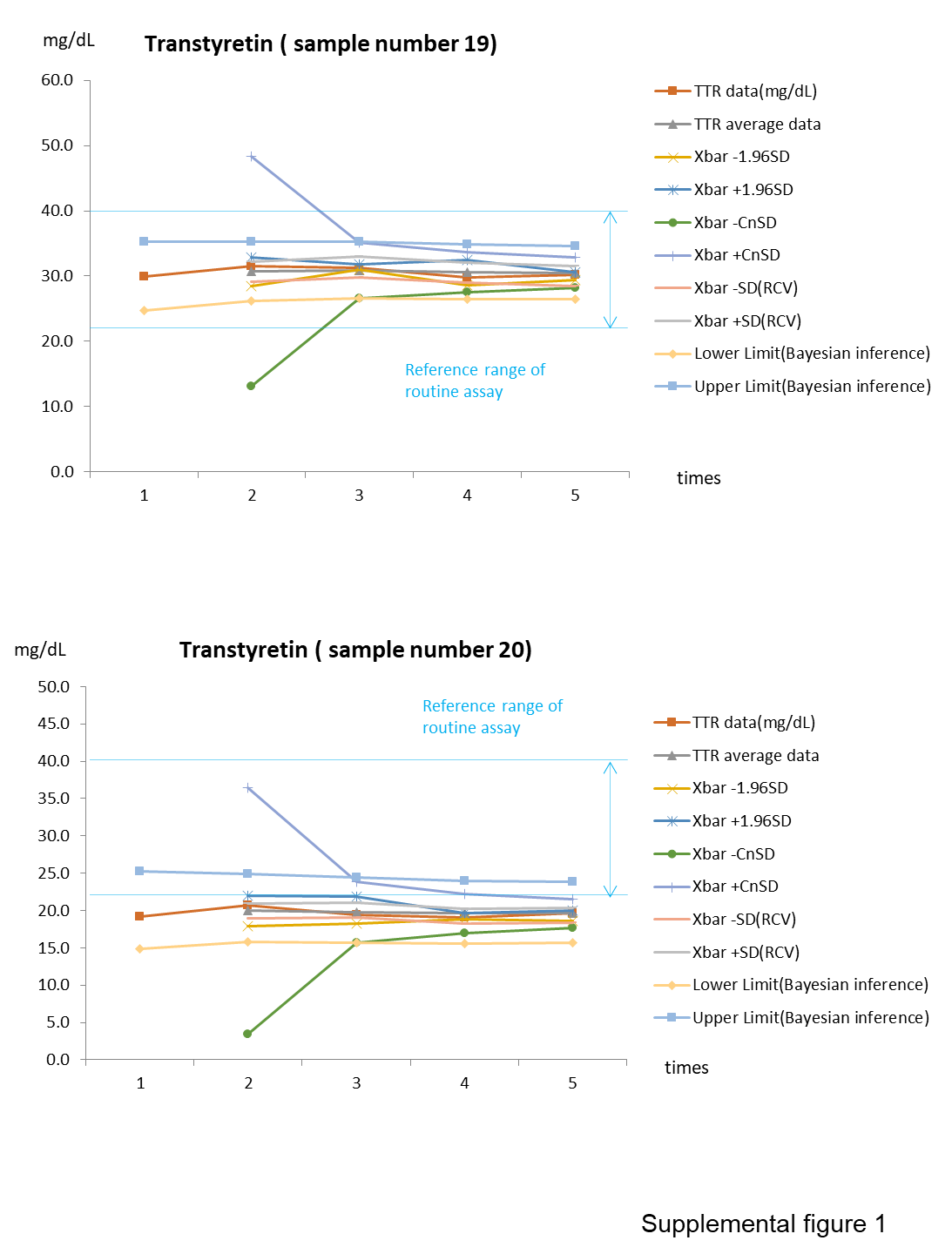


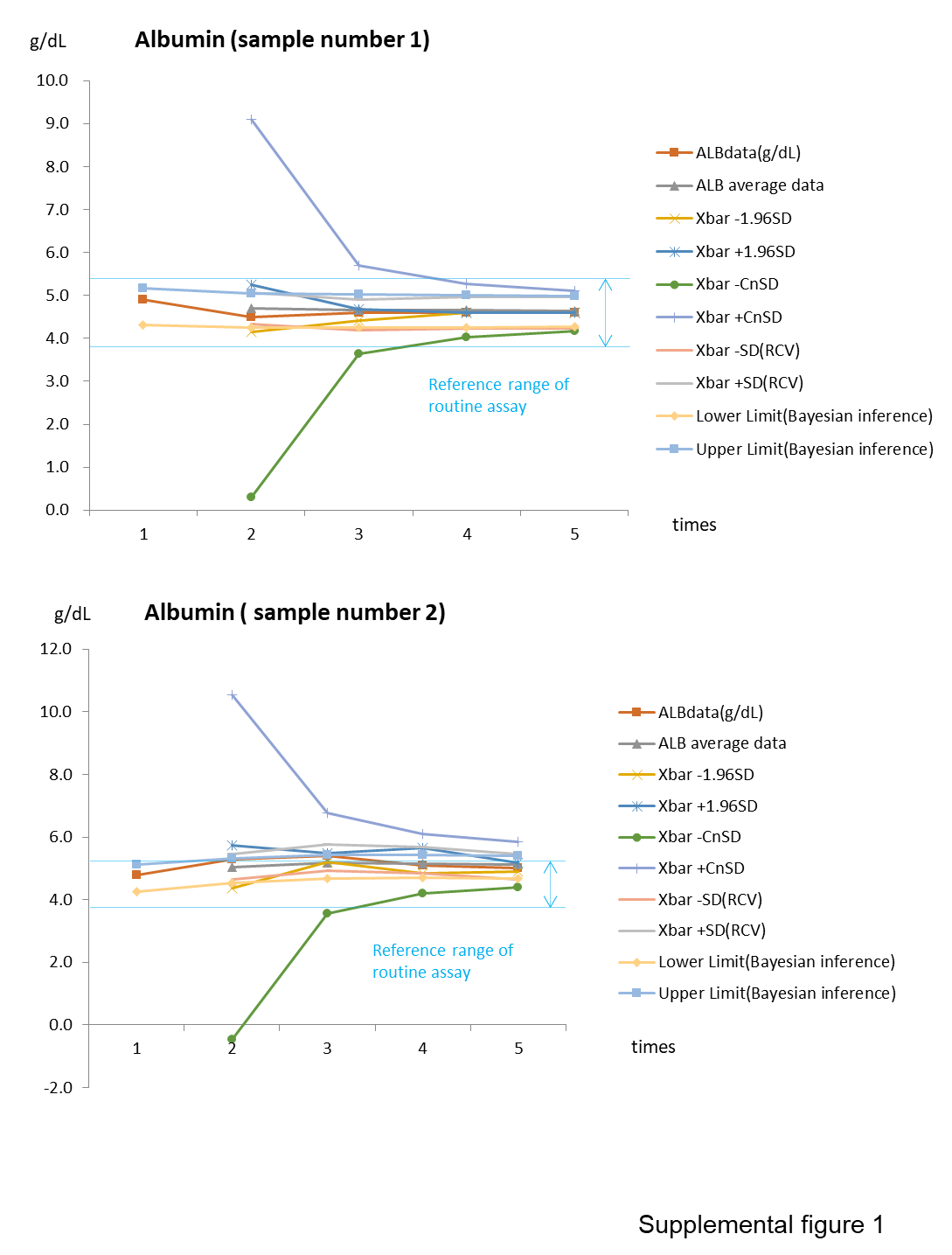


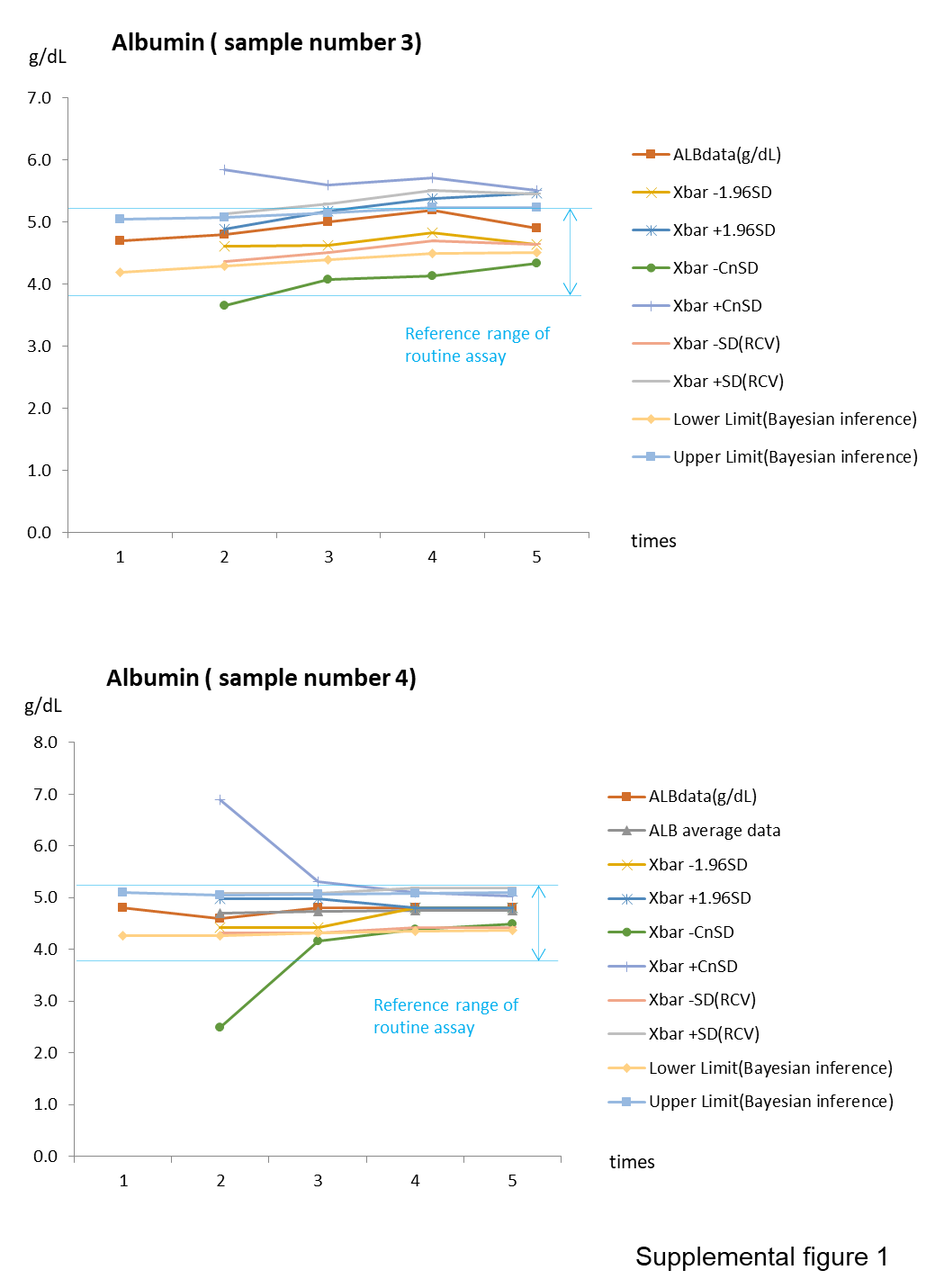


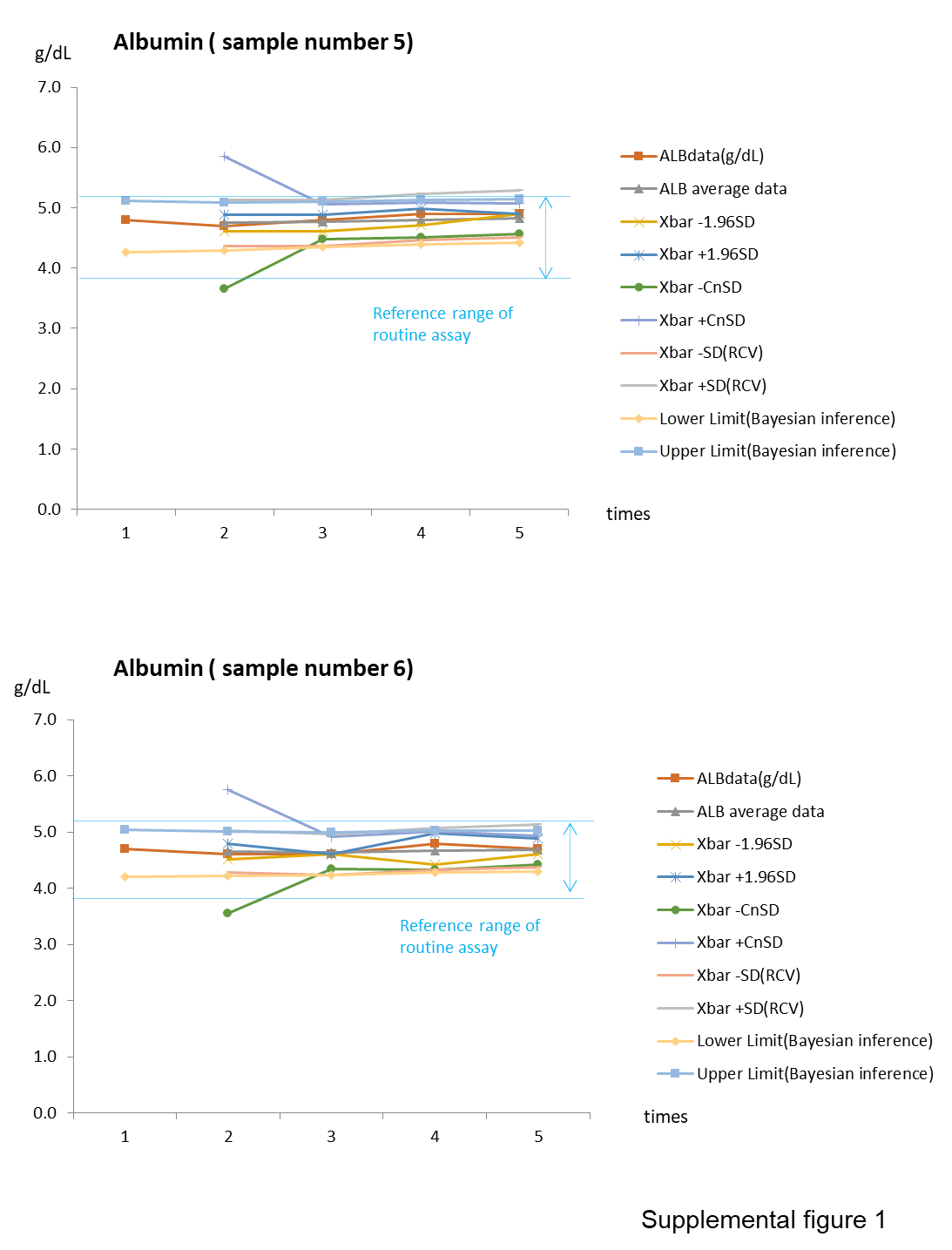


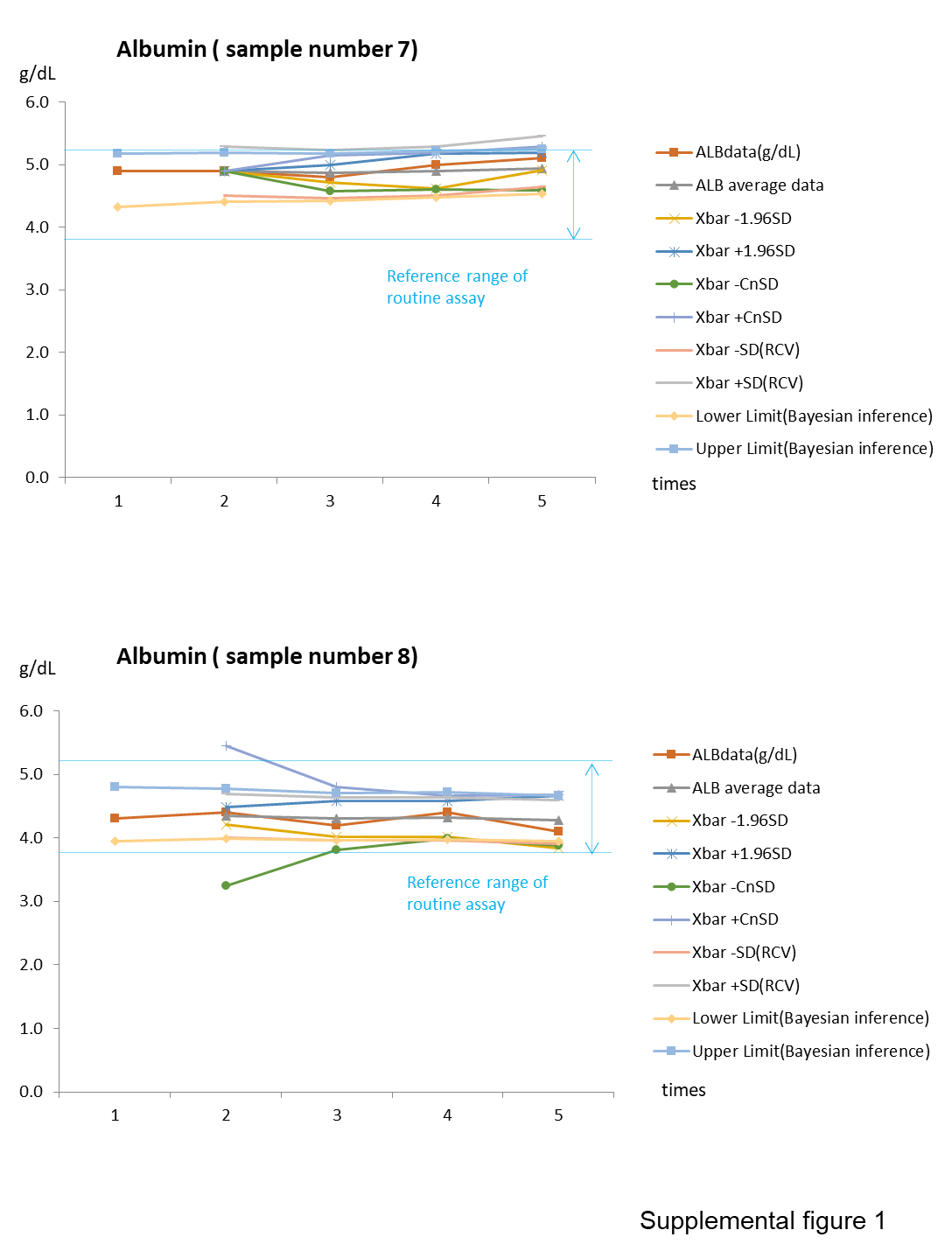


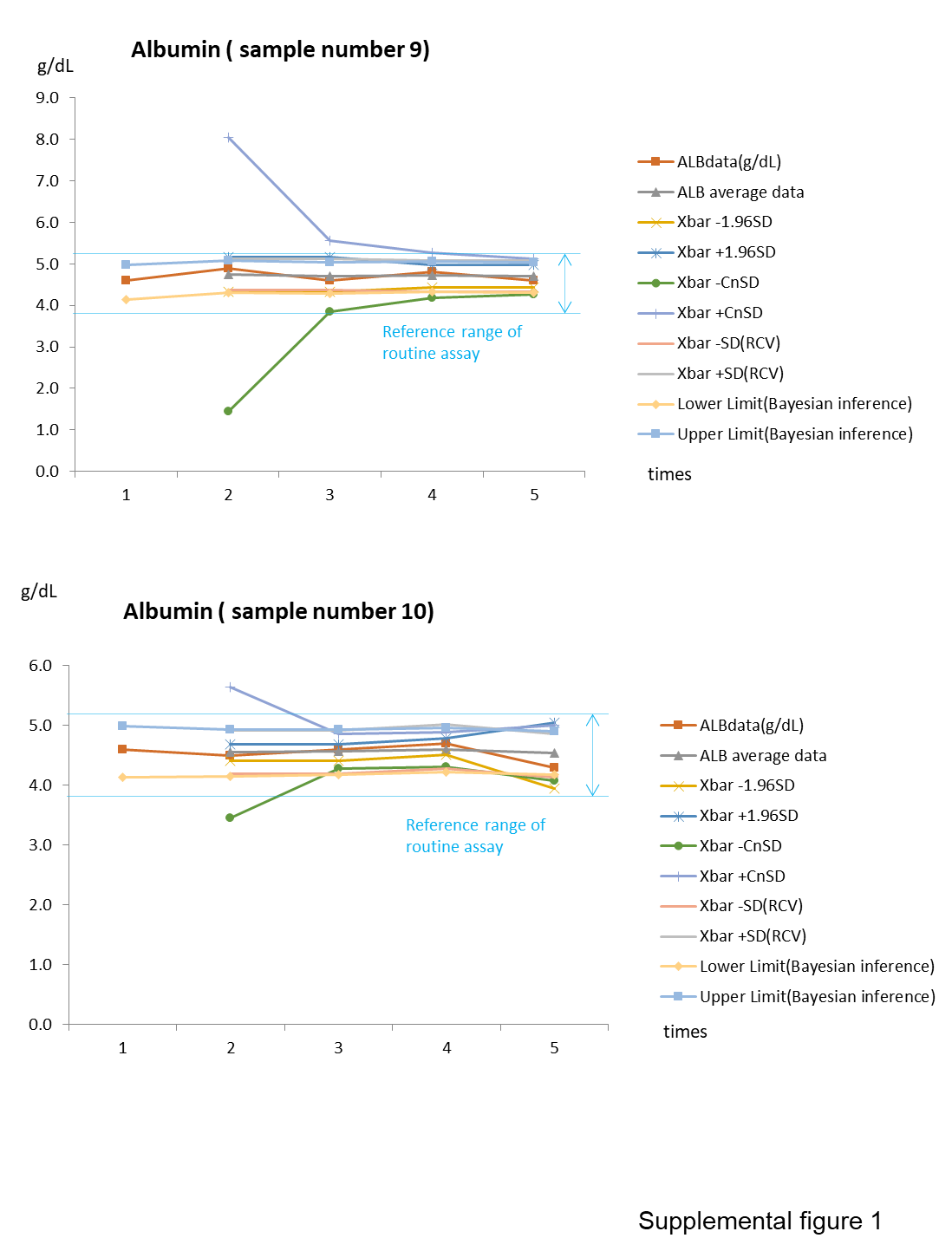


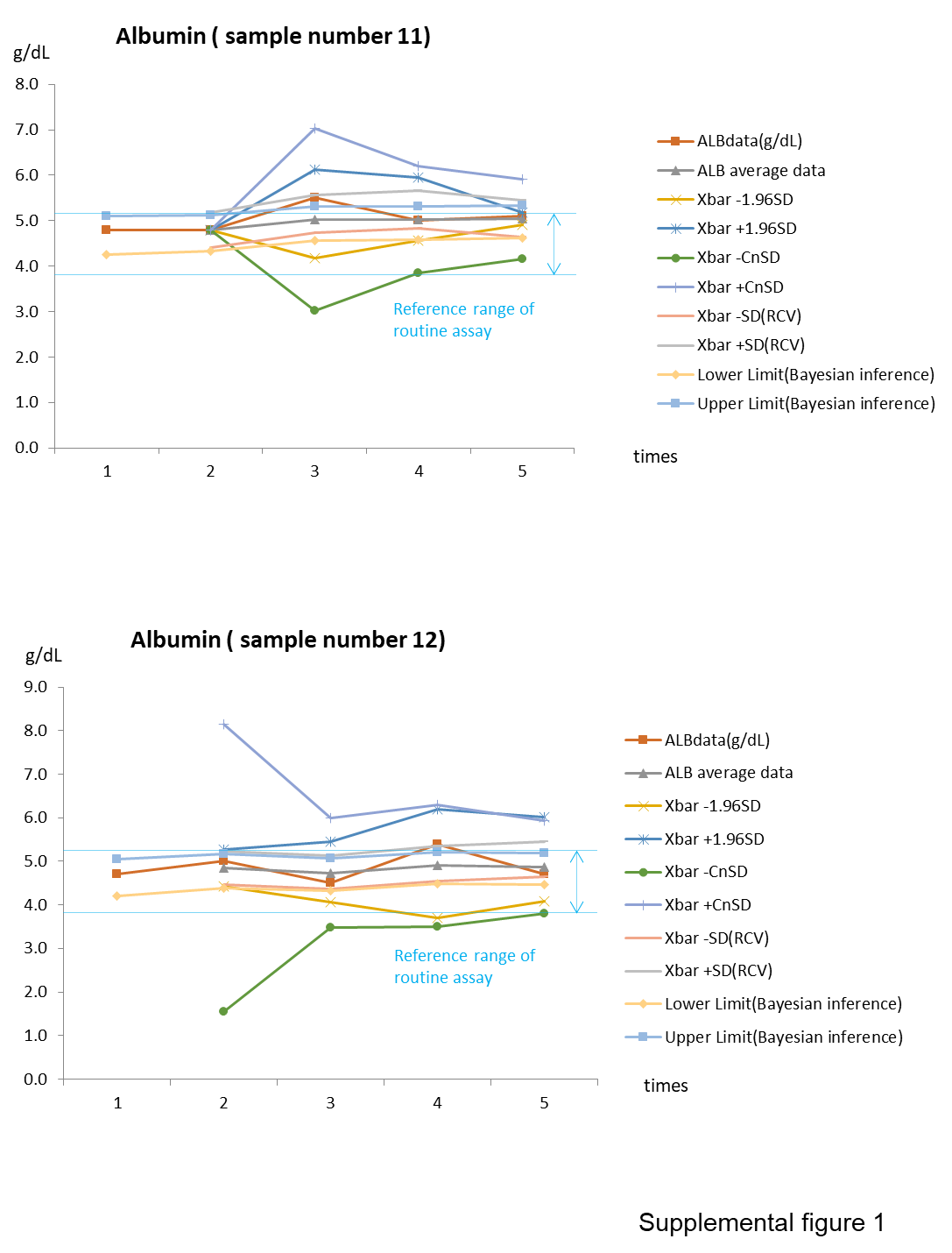


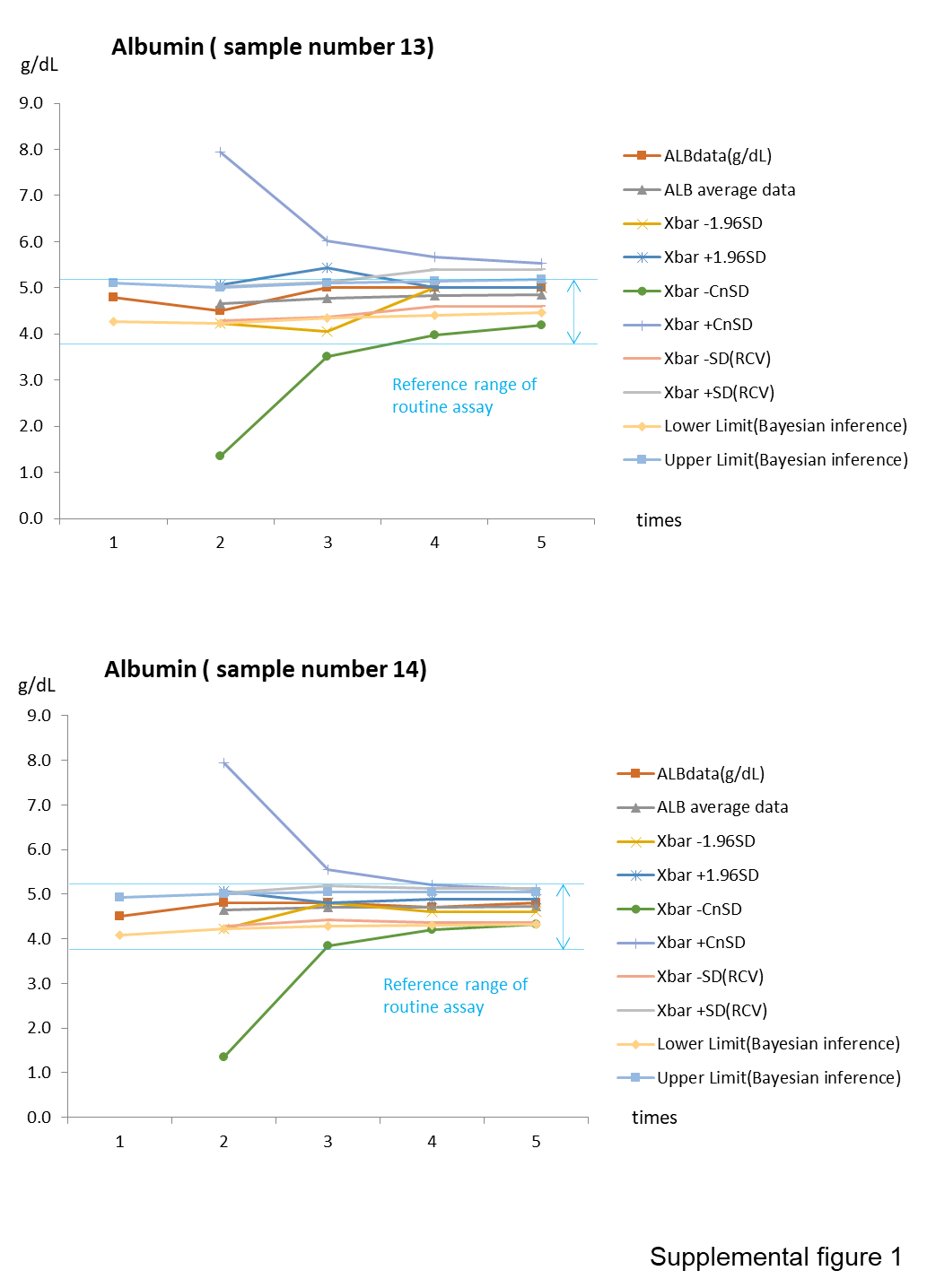


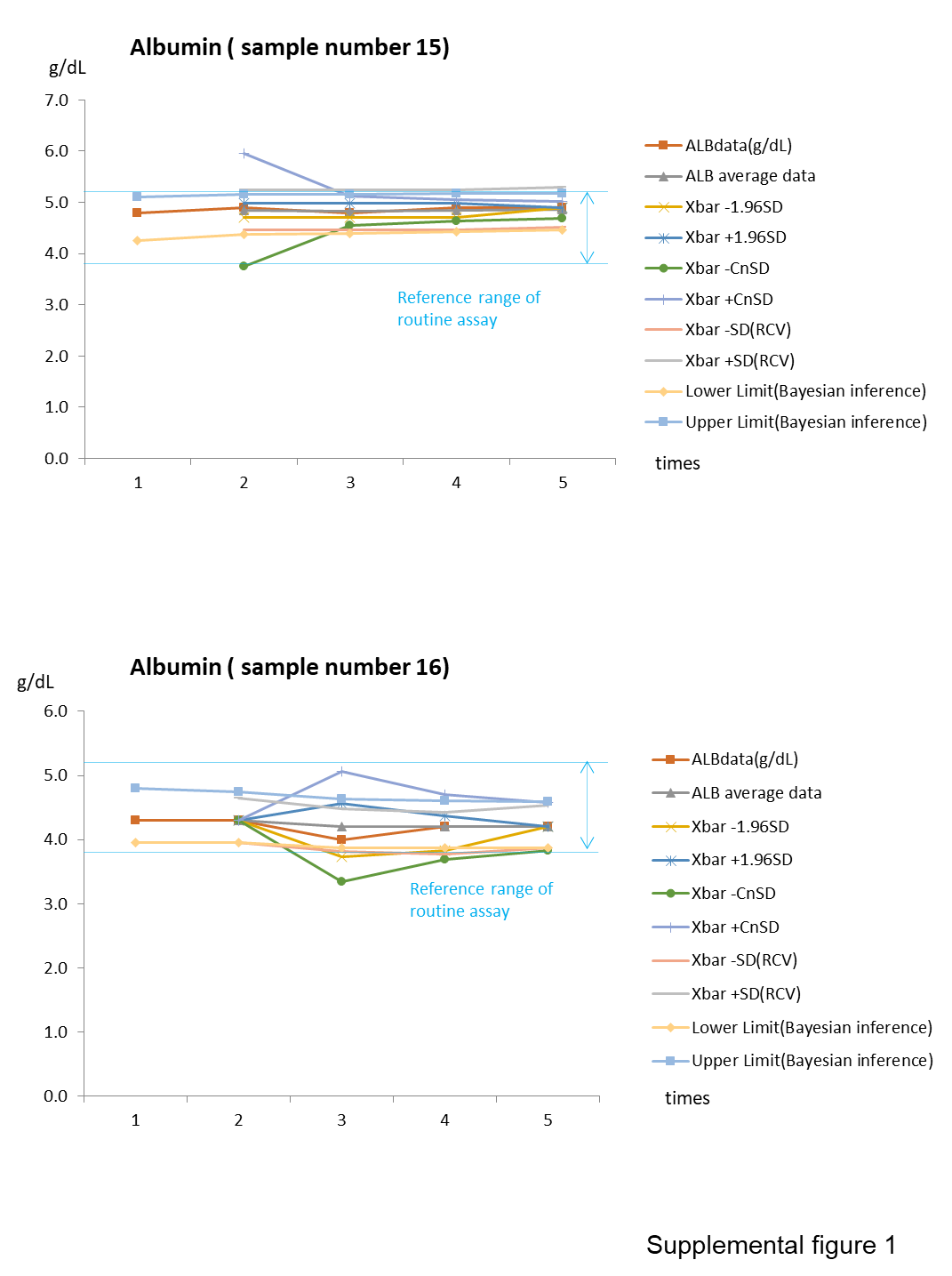


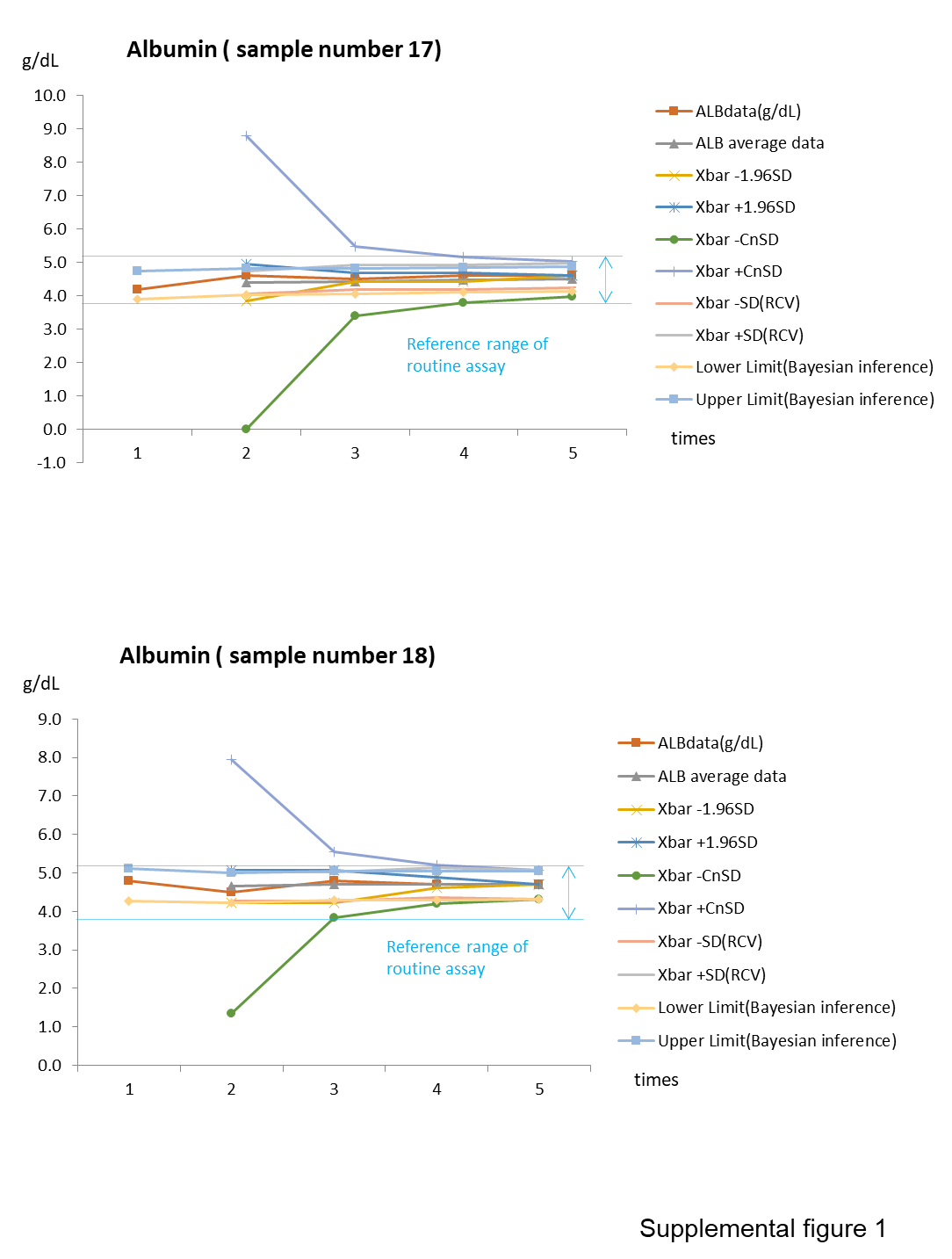


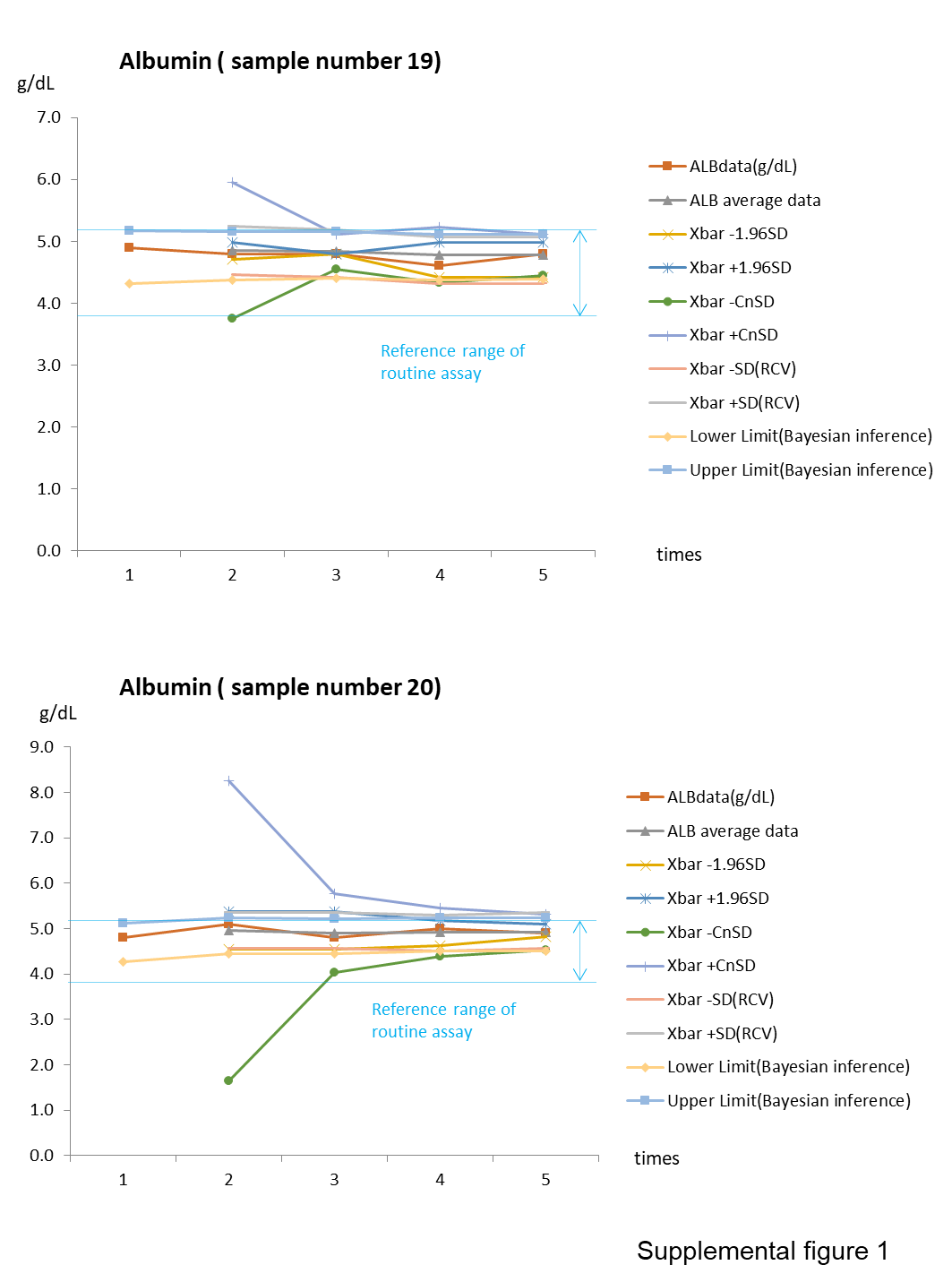

Supplement: Supplementary file 1 — Fig S1 [file JCLA-35-e23639-s001.docx]
