# Supplementary material for: Comparative evaluation of methods to determine intra‐individual reference ranges in nutrition support team (NST)‐related tests
Source: J Clin Lab Anal. 2020 Oct 27;35(2):e23639. doi: 10.1002/jcla.23639 (PMC7891514; doi:10.1002/jcla.23639)
Supplement: Supplementary file 2 — Supplementary Material [file JCLA-35-e23639-s002.docx]

Supplemental figure 1: Temporal changes in measured values for the four test indicators of each subject.

Temporal changes in five measurements of transferrin, albumin, retinol-binding protein, and transthyretin for each of the 20 subjects were shown.

The x-axis shows the number of measurements. The Y-axis shows the measured concentration. The range of the red broken line shows the standard range between individuals used as the routinely analysis. The red square line shows the measured value of each time. The green ▲ line shows the cumulative average of the values measured each time. The purple x line and thin blue ⁎ line show the average value of -1.96 standard deviation (SD) and +1.96SD, respectively. The orange line and the thin purple | line indicate the average value of -Cn x SD and +Cn x SD. The light red line and the light green line indicate the average value-SD (RCV) value and average value + SD (RCV) value, respectively. Thin purple ◆ line and thin blue ■ line show the lower and upper limits of Bayesian inference, respectively. The range among two green lines mean the male reference range of routine assay (see RESULTS 3.2), the range among two red lines mean the female reference range of routine assay (see RESULTS 3.2), and the range among two light blue lines show the total (no gender differences) reference range of routine assay (see RESULTS 3.2).
